# Psychiatric pharmacogenomics: from genetic evidence to clinical integration – structural, educational, and ethical challenges

**DOI:** 10.3389/fphar.2026.1775522

**Published:** 2026-04-07

**Authors:** Andrei Gabriel Mangalagiu, Bogdan Mircea Petrescu, Sorin Riga, Octavian Vasiliu

**Affiliations:** 1 Discipline of Psychiatry II, Department of Clinical Neurosciences, Carol Davila University of Medicine and Pharmacy, Bucharest, Romania; 2 Department of Psychiatry, Dr. Carol Davila University Emergency Central Military Hospital, Bucharest, Romania; 3 Romanian Academy of Medical Sciences, Bucharest, Romania

**Keywords:** antidepressants, antipsychotics, major depressive disorder, mood stabilizers, pharmacogenetics, pharmacogenomics, schizophrenia spectrum disorders, therapeutic guidelines

## Abstract

The role of pharmacogenomics (PGx) for identifying individualized therapeutic approaches in patients with psychiatric disorders is a topic of intense debate in the literature, from clinical, pharmacoeconomic, ethical, educational, and theoretical perspectives. The objectives of this narrative review were (1) to synthesise the genetic evidence base for psychiatric PGx, with particular attention to the hierarchy between pharmacokinetics, pharmacodynamics and emerging epigenetic or polygenic markers; (2) to evaluate the clinical integration of pharmacogenomics in psychiatry, focusing on PGx-guided versus treatment-as-usual randomized trials, meta-analyses, economic evaluations and real-world implementation projects; (3) to analyse structural, educational and ethical challenges that condition the translation of genetic evidence into practice. Based on the reviewed primary and secondary reports, relevant data were found regarding antidepressant PGx, and less for antipsychotics and mood stabilizers. Pharmacoenomic data and structural, economic, and implementation barriers have also been explored, as well as educational and ethical challenges in the field of PGx implementation in psychiatry. In conclusion, psychiatric pharmacogenomics is placed at the intersection between relatively strong but narrow pharmacokinetic evidence, weaker and heterogeneous pharmacodynamic findings, and substantial implementation and ethical constraints. The most clinically actionable data concern CYP2D6 and CYP2C19 variants for certain antidepressants and, to a lesser extent, antipsychotics, which reliably predict serum levels and adverse effects and show modest associations with treatment response and remission.

## 1 Introduction

Although the last decades have witnessed significant progress in the field of psychopharmacology, with the launch of new generations of antipsychotics, new agents for treatment-resistant depression, and advances in the neurobiological understanding of treatment response, psychiatric pharmacotherapy still relies heavily on trial-and-error sequencing, long titration periods, and empirical dose adjustments. Inter-individual variability in efficacy and tolerability is substantial across antidepressants, antipsychotics, and mood stabilisers, and is only partially explained by clinical factors such as diagnosis, symptom profile, or comorbidity (Fornaguera and Miarons, 2025; Grant et al., 2025; Fabbri and Serretti, 2020). Against this background, psychiatric pharmacogenomics (PGx) proposes a mechanistic framework in which inherited and acquired variations in pharmacokinetic (PK) and pharmacodynamic (PD) pathways can be used to individualise drug choice and dosing. The clinical utility of PGx in combination with other strategies, in the psychiatric population, has been examined in the literature, with variable results to date, including outcomes such as symptom severity, adverse events, mental quality of life, polypharmacy, hospitalisations, and readmissions (Bohlen et al., 2023; Tanner et al., 2018; Skokou et al., 2024; Gagiu et al., 2024).

From a genomic perspective, psychiatric disorders are highly polygenic, with hundreds of common variants of very small effect sizes contributing to disease risk (Correia et al., 2022; Santoro et al., 2016). Pharmacogenetic studies broadly recapitulate this architecture: the majority of candidate PD gene variants have weak and inconsistent associations with treatment response, whereas a narrower set of PK genes -particularly CYP2D6 and CYP2C19- show robust links to serum concentrations, adverse events, and, to a lesser extent, clinical outcomes (Fornaguera and Miarons, 2025; Grant et al., 2025; Bousman et al., 2021). These findings are reflected in expert consensus statements and gene–drug-specific dosing guidelines, which converge on a small number of high-confidence interactions (for example, tricyclic antidepressants (TCAs) and several serotonin-selective reuptake inhibitors (SSRIs) in relation to CYP2D6/CYP2C19 phenotype) and remain cautious about most PD markers (Bousman et al., 2021; Beunk et al., 2024).

In addition, a growing body of randomised controlled trials (RCTs) and meta-analyses has evaluated multigene, combinatorial pharmacogenomic panels for major depressive disorder (MDD). These studies and their quantitative syntheses consistently report small-to-moderate improvements in response and remission rates when pharmacogenomic information is available to prescribers, particularly in treatment-resistant populations and in patients with a high burden of predicted gene–drug interactions (Fornaguera and Miarons, 2025; Grant et al., 2025; Brown et al., 2020; Skryabin et al., 2023; Wang et al., 2023; Cheng et al., 2023; Zhang et al., 2025). Yet, effect sizes are modest, heterogeneous and sensitive to study design, industry sponsorship, and adherence to testing recommendations.

Implementation and policy analyses make the situation even more complex. Commentaries and formal evaluations of commercial decision-support tools highlight variability in allele coverage, phenotype calling, and reporting format across panels, as well as concerns about opaque algorithms and sponsorship bias (Bousman and Hopwood, 2016; Bousman et al., 2017; Health Quality Ontario, 2017). Health technology assessments (HTAs) and cost-effectiveness reviews conclude that pharmacogenomic testing can be economically attractive under specific assumptions -test price, prevalence of actionable variants, and effect size- but stop short of recommending routine reimbursement in psychiatry (Health Quality Ontario, 2017; Morris et al., 2022). Population-genetic work shows substantial ancestry-related variation in CYP2D6/CYP2C19 phenotypes, raising doubts about the direct transferability of predominantly European-derived panels to under-represented populations and low- and middle-income countries (LMICs) (Koopmans et al., 2021).

Finally, hospital-based and primary-care implementation projects demonstrate that pharmacogenetic testing can be integrated into electronic health records (EHRs) with decision support, but only where substantial infrastructural investment, informatics support, and clinician education are available (Wu et al., 2025; Ginsburg et al., 2021; Cavallari et al., 2017; Maruf et al., 2020; Bhimpuria, 2024; Rollinson et al., 2020; Lara et al., 2021). These realities foreground structural, educational, and ethical questions that go well beyond the narrow issue of whether particular gene–drug pairs are “clinically significant”.

In this context, the present narrative review has three aims: (1) to synthesise the genetic evidence base for psychiatric pharmacogenomics, with particular attention to the hierarchy between PK, PD and emerging epigenetic or polygenic markers; (2) to evaluate the clinical integration of pharmacogenomics in psychiatry, focusing on PGx-guided versus treatment-as-usual (TAU) RCTs, meta-analyses, economic evaluations and real-world implementation projects; (3) to analyse structural, educational and ethical challenges that condition the translation of genetic evidence into practice, including guideline heterogeneity, health-system infrastructure, ancestry and equity, informed consent, but also the role of commercial interests.

Rather than treating pharmacogenomics as an all-or-nothing innovation, we argue for a more nuanced view: psychiatric pharmacogenomics is best understood as an incremental optimisation tool whose clinical value depends critically on how it is embedded within wider systems of care, education, and regulation (Fornaguera and Miarons, 2025; Grant et al., 2025; Bousman et al., 2021; Brown et al., 2020; Skryabin et al., 2023; Wang et al., 2023; Cheng et al., 2023; Zhang et al., 2025; Vasiliu, 2023). We also considered that focusing on certain classes of psychotropics would be limiting and endanger the generalisability of the results; therefore, we did not restrict the review to specific pharmacological agents or nosological indications (Vasiliu et al., 2017; American Psychiatric Association, 2022).

## 2 Methods

This is a narrative, non-systematic review of psychiatric pharmacogenomics. The objective was not to exhaustively capture every published genetic association, but to integrate thematically the most informative and methodologically robust evidence across three domains: (i) genetic architecture and pharmacogenetic markers; (ii) clinical integration through PGx-guided vs. TAU primary and secondary reports; and (iii) implementation-related structural, educational, and ethical issues. Given the rapidly evolving literature and the heterogeneity of study designs, a narrative approach was considered more appropriate than a fully systematic review.

### 2.1 Sources and search strategy

The evidence base integrates literature identified through searches in PubMed/MEDLINE, Scopus, and Web of Science/Clarivate, complemented by backward and forward citation tracking of key reviews, consensus statements, guidelines, and HTA reports. Database searches yielded 262 records (PubMed n = 58; Scopus n = 105; Web of Science n = 99). After de-duplication, 129 unique records remained and constituted the initial seed set for the narrative synthesis. Supplementary Figure 1 provides a PRISMA-style flow schematic summarizing record identification, de-duplication, and theme-driven inclusion/mapping for transparency. Searches covered publications from January 2015 to August 2025 and combined controlled vocabulary and free-text terms related to (a) population/indication: depression, major depressive disorder, bipolar disorder, schizophrenia, psychosis, anxiety disorders, post-traumatic stress disorder, obsessive-compulsive disorder, autism spectrum disorder, intellectual disability, substance use disorders; (b) intervention/exposure: pharmacogenetic, pharmacogenomic, genetic, CYP2D6, CYP2C19, pharmacokinetic, pharmacodynamic, polygenic, epigenetic; (c) comparator: treatment-as-usual, standard of care, usual care, non-guided, unguided; (d) outcomes: response, remission, adverse drug reaction, tolerability, side effects, hospitalisation, cost-effectiveness, cost–utility, implementation, decision support.

We prioritised studies explicitly labelled as pharmacogenetic/pharmacogenomic and excluded purely diagnostic, risk, or endophenotype genetics unless they directly informed treatment response or tolerability.

### 2.2 Study selection and eligibility

However, selection was driven by relevance to the three focal domains rather than by a rigid PICOS template, to increase the likelihood of collecting more data to support the previously formulated objectives.

Inclusion strategies were determined by the *research design*, specifically RCTs and patient- or rater-blinded studies comparing PGx-guided versus unguided prescribing in psychiatric or closely related populations; systematic reviews and meta-analyses of pharmacogenetic markers in antidepressant, antipsychotic and mood-stabiliser treatment; consensus statements, guideline documents and HTAs addressing psychiatric pharmacogenomics or psychotropic gene–drug pairs; implementation studies and scoping reviews of PGx testing in hospital, primary care and community pharmacy settings; economic evaluations and simulation models assessing the cost-effectiveness of PGx-guided prescribing; narrative reviews and overviews of reviews providing higher-level syntheses of PGx evidence in psychiatry and neurology. Also, the selected criteria referred to the *populations*, i.e., adults were the primary focus, with inclusion of paediatric and older-adult studies where they directly informed translational questions (for example, antidepressant PGx in youth or late-life depression). *Interventions and markers* allowed for inclusion in the current review referred to gene–drug pairs with guideline-level or emerging evidence (for example, CYP2D6/CYP2C19 and antidepressants/antipsychotics); multigene combinatorial panels used in clinical trials or implementation projects; pharmacodynamic markers and exploratory epigenetic/transcriptomic signatures where they informed the broader hierarchy of evidence. The targeted *outcomes* were clinical response/remission, adverse events, treatment persistence, hospitalisation, healthcare utilisation, cost-effectiveness, and implementation metrics (for example, uptake, clinical decision support (CDS) alert firing, prescriber adherence).

The formulated exclusion criteria were related to *design*, specifically case reports and small uncontrolled case series unless they illustrated unique ethical or implementation issues, but also to *intervention*, i.e., studies focusing exclusively on non-psychiatric drugs outside supportive care or clear psychotropic relevance (for example, oncology PGx with no psychiatric outcomes), and *outcomes*-genetic studies without treatment or tolerability outcomes. Regarding the *population*, animal studies were excluded from analysis; also, studies focused only on pediatric and geriatric populations, from which no relevant data regarding PGx variables could be derived for the adult population.

Given the narrative design, selection was iterative: as thematic patterns emerged, additional targeted searches were performed (for example, for HTAs, national landscape reviews, or specific guideline bodies).

### 2.3 Data extraction and synthesis

For RCTs and meta-analyses of PGx-guided versus TAU antidepressant treatment, we extracted: sample size, population (for example, general MDD, treatment-resistant, elderly, adolescents), panel characteristics (genes included, algorithm type), primary and secondary outcomes, effect sizes, and key risk-of-bias issues. For pharmacogenetic meta-analyses of individual markers, we focused on: gene(s) studied, drug classes, outcome domains (response, remission, side effects), pooled effect sizes, and heterogeneity metrics. For guidelines, consensus statements, and HTAs, we abstracted the scope of recommendations, strength and grading of evidence, genes and drugs covered, and explicit caveats (e.g., ancestry, effect size, cost-effectiveness). Implementation studies and health-system reports were reviewed for details on: testing strategy (pre-emptive versus reactive), integration into EHR/CDS, funding and reimbursement models, clinician education, and reported facilitators/barriers.

Synthesis was thematic rather than quantitative. *First*, we organised the genetic evidence into a hierarchy of PK, PD, and exploratory markers, contrasting antidepressant and antipsychotic data with sparser evidence for mood stabilisers and other indications. *Second*, we summarised clinical integration data by type of evidence: RCTs and meta-analyses, economic evaluations, and real-world implementation programmes. *Third*, we mapped structural, educational, and ethical issues identified in guidelines, HTAs, and implementation reports, with particular attention to ancestry, equity, consent, data governance, and commercial interests. No formal risk-of-bias tool was systematically applied; instead, we used domain-informed credibility checks (AMSTAR-2 domains for meta-analyses and standard RCT bias domains) and preferentially weighted larger blinded RCTs, independent meta-analyses, and non-industry HTAs when interpreting the evidence.

Given the narrative design, we did not formally quantify the overlap of primary studies across included systematic reviews and meta-analyses. Therefore, some duplication of primary evidence across secondary sources is possible.

## 3 Results

The articles reviewed in this section represent 34 clinical studies (Table 1), 50 reviews and meta-analyses (Table 2), and 8 consensus papers, guidelines, and other sources (Table 3). Nine additional studies are cited and discussed in the main text where they inform specific mechanistic, methodological, or contextual points, but are summarized separately in Table 4. The data were distributed across distinct chapters, addressing the objectives of the review: the effects of PGx testing on PK and PD parameters in psychopharmacology, the clinical impact, and recommendations for PGx in this field, as well as the challenges in the real-life implementation of PGx.

**TABLE 1:** Clinical trials exploring the role of PGx in psychopharmacology.

Ref	Authors	Country/Region (participants)	Setting (as reported)	Methodology	Participants and intervention	Outcomes	Results	Conclusions
Antidepressants — PK/PD and observational clinical studies								
35	Berm et al. (2016)	Netherlands	Inpatient	Observational PK study	81 subjects (41 on nortriptyline, 40 on venlafaxine), population not restricted to an MDD diagnosis	Serum concentrations of the active drugs	CYP2D6 genotype (3/4-based) predicts drug levels: PM/IM genotypes showed more supratherapeutic nortriptyline concentrations; PM status was also associated with higher risk of non-response on HAM-D, but not MADRS	PGx is a valuable tool in addition to TDM to prevent supratherapeutic drug levels in elderly patients with a PM genotype
36	Aldrich et al. (2019)	USA	Inpatient psychiatric unit	Observational PK study	263 patients, anxiety and/or depressive disorders, age < 19 years, treated with citalopram/escitalopram	Tolerability and response to (es)citalopram	CYP2C19 PMs had more ADRs	Youth mirrors adult data
38	Yuce-Artun et al. (2016)	Turkey	Outpatient psychiatry unit (university hospital)	Observational PK study	50 Turkish MDD patients receiving sertraline	Drug levels of the parent drug and its metabolite, N-desmethyl sertraline	CYP2B6/CYP2C19 polymorphisms affect sertraline metabolism	CYP2B6*6 and *9 variant alleles had a significant decreasing effect on sertraline metabolism
43	Yin et al. (2016)	China (Chinese Han origin)	Clinical psychiatry recruitment (setting NR: inpatient/outpatient)	Candidate-gene association RCT	290 Chinese MDD patients undergoing SSRI treatment	AD effects in relation to the DRD4 polymorphisms	The frequency of the DRD4 rs1800544 CG genotype was significantly higher in responders than in nonresponders	Polymorphisms of the DRD4 gene appear to be associated with SSRI treatment response in Chinese MDD patients
44	Yin et al. (2015)	China (Chinese Han origin)	NR	Candidate-gene association RCT	290 Chinese MDD patients, SSRI treatment	AD and DRD4 polymorphisms, but also cortisol, ACTH, TSH, T3, T4, FT3, fT4 levels	Mixed COMT/DRD findings	The combination of all neuroendocrine factors, clinical characteristics and gene polymorphisms predicted 74.8% of the variation in SSRI response and 65.5% in SSRI remission
46	Yeh et al. (2015)	Taiwan (Taipei)	Mixed	Candidate-gene naturalistic follow-up study	557 MDD patients, AD and NGF polymorphisms	AD and NGF polymorphisms	NGF variants are indirectly associated with remission rates after 8-week AD treatment	Psychological mediators (e.g., “harm avoidance”) were identified in the NGF–remission association
47	Kato et al. (2015)	Japan	Inpatient	Pooled RCT genetic analysis	168 Japanese MDD patients	AD treatment (paroxetine, fluvoxamine, milnacipran) and polymorphisms of multiple genes (5-HTTLPR, FGF2, HTR1A, ADRA2A, etc.)	EPI ≥20% reduction in HAM-D score by week 2 predicted treatment outcome, and specific genetic variants predicted outcome only when combined with EPI	Combining EPI with genetic variants improves the prediction of AD treatment outcome compared with genetics alone
49	Bruzzone et al. (2025)	Denmark (Capital Region)	NR (clinical trial; inpatient/outpatient not specified)	Epigenetic cohort study	90 unmedicated adults with MDD, treatment with escitalopram	Clinical AD response measured by change in HAMD-17	Baseline TPH2 methylation was associated with clinical response and change in depressive symptoms at 8 weeks. No strong predictive value for SLC6A4 or TPH2 DNA methylation as clinical biomarkers (AUC values for prediction were modest). Methylation changes at SLC6A4 and TPH2 were observed at the trend level over 12 weeks.	TPH2 and SLC6A4 methylation patterns may inform associations with AD response, but are unlikely to serve as reliable clinical predictors on their own
51	Poweleit et al. (2019)	USA	Inpatient psychiatry service + outpatient follow-up	Retrospective cohort PGx analysis	352 pediatric patients, sertraline treatment	Sertraline dose during the titration phase, treatment response dose, tolerability, and adverse events	CYP2C19 reduced-function alleles were associated with lower maximum sertraline dose during titration. Predicted SLC6A4 expression levels were associated with duration of sertraline treatment. Combinatorial analyses suggested that PD genes and clinical factors influence dosing and response.	Both PK factors (CYP2C19) and PD genetic variants (HTR2A, SLC6A4) - along with clinical/demographic components - may influence sertraline dosing, response, and tolerability in pediatric anxiety and depressive disorders.
52	Amitai et al. (2016)	Israel	NR	OL, clinical PGx association study	87 outpatients, 7-18 years of age, diagnosed with MDD, dysthymia, and/or anxiety disorders, receiving citalopram treatment	Association of specific genotypes with citalopram-related side effects, especially agitation as reported during treatment.	Agitation was more common in boys than in girls during citalopram treatment. Subjects with the 5-HTR1Dβ CC genotype reported higher rates of agitation than the CG and GG genotype groups	The 5-HTR1Dβ polymorphism appears to be associated with citalopram-related agitation in children and adolescents treated for depression and/or anxiety
53	Honeycutt et al. (2024)	USA	Outpatient	Prospective, OL, PGx/PK pilot study	Pediatric population (12-17 years), escitalopram treatment	PK parameters, adverse events	CYP2C19 polymorphisms affect escitalopram PK (AUC, Ctrough, t1/2) and the risk of certain adverse events.	CYP2C19 phenotype influences escitalopram PK. Slower CYP2D6 metabolism was associated with ↑ akathisia, and HTR2A A/A or A/G genotypes were associated with ↑ risk of self-harm or harm to others.
84	Müller et al. (2024)	Germany (multicenter; EMC trial)	NR	Prospective observational cohort study	∼2200 MDD patients, predominantly of European ancestry	1) Psychiatric disorder risk (genetic liability); 2) AD treatment response; 3) EMC as a clinical marker	PRS were significantly associated with psychiatric disorder risk and likelihood of early medication change, but not associated with AD treatment response	Common polygenic variation contributes to disease liability and early treatment instability, but does not meaningfully predict AD response, highlighting the limits of PRS-based precision prescribing at present
Antidepressants — PGx-guided RCTs and pragmatic trials								
87	Greden et al. (2019)	USA	Outpatient	RCT	1167 MDD patients, PGx-guided treatment vs. TAU	Symptom improvement on the HAM-D17 at 8 weeks; response and remission rates	The primary endpoint (mean symptom improvement) did not differ significantly between groups. However, the PGx-guided group showed significantly higher response and remission rates compared with TAU, particularly among patients whose baseline medications were genetically incongruent.	PGx-guided care modestly but significantly improved response and remission rates in MDD compared with usual care
88	Oslin et al. (2022)	USA	Outpatient (VA system)	Pragmatic RCT	1944 MDD patients, PGx testing for DGI vs. usual care	Remission of depressive symptoms at 24 weeks (defined by PHQ-9 score)	PGx testing significantly reduced prescribing of medications with predicted DGI. Remission rates were modestly but significantly higher in the PGx-guided group at earlier time points; the difference at 24 weeks was small and of borderline clinical significance.	PGx testing improved medication selection and showed small improvements in remission
89	Thase et al. (2019)	USA	Outpatient	RCT	912 MDD patients, PGx-guided treatment using combinatorial PGx test vs. TAU	Symptom improvement at 8 weeks, response, remission, and tolerability outcomes	In this DGI subgroup, PGx-guided care resulted in significantly greater symptom improvement, higher response and remission rates, and higher overall treatment success compared with treatment as usual. Effects were larger than those observed in the full GUIDED cohort.	PGx-guided treatment provides greater clinical benefit for patients who are prescribed medications with actionable DGI
90	Bradley et al. (2018)	USA	Outpatient	RCT	316 patients with MDD and/or anxiety disorders, targeted PGx-guided treatment using a combinatorial PGx test vs. TAU	Change in depressive and anxiety symptoms severity at week 8; response, remission, tolerability and healthcare utilisation	The PGx-guided group showed significantly greater symptom improvement, as well as higher response and remission rates, compared with treatment as usual. Benefits were strongest among patients whose baseline medications were genetically incongruent with their genetic profiles.	Targeted PGx-guided prescribing significantly improved clinical outcomes for patients with depression and anxiety, supporting the clinical utility of PGx testing
91	Tiwari et al. (2022)	Canada	Clinical settings (psychiatric + primary care); inpatient/outpatient NR	RCT	∼300 MDD patients, combinatorial PGx–guided treatment versus TAU	Change in depressive symptoms at 8 weeks; response, remission, functional improvement, and tolerability	The PGx-guided group demonstrated significantly higher response and remission rates compared with TAU. Mean symptom improvement favored the PGx-guided arm, with effect sizes similar to other combinatorial PGx trials. Benefits were greatest among patients with baseline gene–drug incongruence.	Combinatorial PGx testing improved clinical outcomes in MDD in a Canadian setting, supporting its clinical utility.
92	Minelli et al. (2021)	Italy (Brescia)	Outpatient	Trial design / protocol paper	∼300 patients planned, predominantly of European ancestry, diagnosed with MDD, combinatorial PGx-guided treatment vs. TAU	Change in depressive symptoms; response, remission, functional outcomes, and tolerability	NR (results not reported in this publication)	Describes rationale and methods for a participant- and rater-blinded trial (PANDORA).
93	Shan et al. (2019)	China (Han population)	Mixed	RCT	72 Chinese patients with MDD, combinatorial PGx AD treatment vs. TAU	Change in depressive symptom severity (HAMD) over 8 weeks; response, remission, time to improvement, ADRs	The PGx-guided group showed greater reduction in depressive symptoms and higher response rates compared with TAU. Differences in remission rates favored the PGx-guided arm but were limited by small sample size.	This preliminary randomized study suggests that combinatorial PGx testing may improve AD treatment outcomes in MDD.
94	Forester et al. (2020)	USA	Outpatient	RCT	∼300 older adults (≥60 years) with MDD, combinatorial PGx AD treatment vs. TAU	Depressive symptom improvement at week 8; response, remission, cognitive and functional outcomes, and tolerability in relation to a combinatorial PGx panel	Older adults receiving PGx-guided care showed significantly greater symptom improvement, as well as higher response and remission rates, compared with TAU. Benefits were most pronounced in patients whose baseline medications had predicted DGI.	Combinatorial PGx testing improves clinical outcomes in late-life depression, supporting its utility in geriatric populations where polypharmacy and altered drug metabolism are common.
95	Vande Voort JL et al. (2022)	USA	Mixed	RCT	176 adolescents (ages 13-18) with MDD, combinatorial PGx AD treatment vs. TAU	Change in depressive symptom severity at 8 weeks (CDRS-R) in relation to a combinatorial PGx panel	The primary outcome (mean symptom improvement) did not differ significantly between PGx-guided care and TAU. Response and remission rates were numerically higher in the PGx-guided group, but the differences were not statistically significant.	In adolescents with MDD, combinatorial PGx testing did not significantly improve depressive symptoms compared with usual care. Findings contrast with adult trials and highlight potential age-related differences in PGx utility.
100	Vos et al. (2025)	Netherlands	NR	RCT	200 MDD patients, predominantly of European ancestry (Dutch clinical setting); PGx-based dosing (CYP2D6/CYP2C19) vs. phenotype-based dosing (therapeutic drug monitoring-guided) vs. standard dosing	Time to reach therapeutic nortriptyline plasma concentration	Both PGx-based and phenotype-based dosing achieved therapeutic concentrations faster and more accurately than standard dosing. Phenotype-based dosing showed the highest precision, while PGx-based dosing reduced early under- and overdosing risk.	Individualized dosing strategies outperform standard dosing for nortriptyline. PGx guidance is particularly valuable early in treatment, while phenotype-based approaches optimize longer-term dose precision.
Antipsychotics and mood stabilizers — clinical studies								
41	Li et al. (2019)	USA	Inpatient	Candidate-gene association	171 male patients with schizophrenia/schizoaffective disorder of European or African ancestry	Symptom change and 5HTR2C polymorphisms	A -759C-Ser23 haplotype was similarly associated with positive and negative symptom improvement	5HTR2C variants have a modest effect on antipsychotics with 5HTR2C antagonism/inverse agonism
65	Zhang et al. (2015)	USA + Canada (multisite)	NR	Prospective cohort PGx association study	327 patients with FEP, receiving antipsychotics	Antipsychotic treatment response measured by changes in symptom severity scales (positive, negative, total psychopathology) over 6–12 weeks; DRD2 rs2514218 risk variant	The DRD2 risk allele (C) at rs2514218 was associated with greater improvement in total psychopathology compared with the T allele. Similar associations were observed for improvement in positive symptoms. Effect sizes remained significant after controlling for covariates (age, sex, treatment type).	The DRD2 schizophrenia risk variant (rs2514218) is associated with antipsychotic treatment response in first-episode psychosis, suggesting that genetic variation at the DRD2 locus may contribute to interindividual differences in clinical outcomes.
72	Creta et al. (2015)	USA (STEP-BD; NIMH-funded)	Outpatient	Observational genetic association study	486 patients with bipolar disorder, receiving atypical antipsychotics and mood stabilizers	Weight gain induced by psychotropics and the relation to SNPs of 16 candidate genes	No association was found between phenotypes and individual polymorphisms or pathways after multiple-test correction. HTR2C, LEP, FTO, and TBC1D1 represented the top genes for weight gain during treatment with the two categories of psychotropics.	Individual genes probably play a limited role in psychotropic-induced weight gain.
98	Jürgens et al. (2020)	Denmark (Capital Region)	Outpatient	Pragmatic RCT	1998 patients, predominantly of European ancestry (Danish national cohort), adults with SSD; routine CYP2D6 and CYP2C19 genotyping with results available to clinicians at treatment initiation	Antipsychotic drug persistence (time to discontinuation or switch) over follow-up in relation to CYP2D6 and CYP2C19 genotypes	Routine CYP2D6/CYP2C19 genotyping did not significantly improve overall antipsychotic drug persistence compared with usual care.	In routine clinical practice, pre-emptive CYP2D6 and CYP2C19 genotyping alone did not improve antipsychotic treatment persistence in patients with schizophrenia.
Implementation, economics and real-world prevalence								
79	de Miguel et al. (2023)	Spain (Santa Faz, Alicante)	NR	Implementation pilot	33 patients with ASD, receiving psychotropics	Frequency and severity of psychotropic-related adverse drug reactions before vs after PGx-guided intervention	Implementation of PGx guidance was associated with a reduction in ADR, fewer severe side effects, and improved treatment tolerability in most participants	PGx testing may help prevent or reduce psychotropic adverse events in ASD
97	Dorfman et al. (2020)	Canada (Toronto, Ontario)	Long-term care	Observational, retrospective cohort study	∼1000 long-term care residents, older adults with depression, chronic pain, and/or dementia; individualised medication management program	Changes in medication burden, symptom control (depression, pain, behavioral symptoms of dementia), and adverse drug events	Implementation of individualized medication management was associated with reduced polypharmacy, improved symptom management, and fewer potentially inappropriate medications. Benefits were observed across depression, pain, and dementia-related outcomes.	Individualized medication management, incorporating PGx information within a multidisciplinary framework, can improve clinical outcomes in long-term care residents, particularly in populations at high risk for adverse drug events.
99	Ter Hark et al. (2025)	Netherlands	Multicenter RCT (Netherlands); inpatient/outpatient NR	RCT	220 MDD patients, genotype-specific dosing of TCA guided by PGx testing	ICER, typically expressed as cost per QALY gained; depression symptom improvement, ADR, treatment discontinuation	Genotype-guided TCA dosing resulted in fewer adverse drug reactions and improved tolerability. From a healthcare payer perspective, PGx-guided dosing was cost-effective or cost-saving compared with standard dosing, depending on willingness-to-pay thresholds.	Genotype-specific dosing of tricyclic ADs is economically favorable in patients with MDD, supporting the implementation of CYP2D6/CYP2C19-guided dosing strategies in routine care.
101	Jablonski et al. (2020)	USA	NR	Retrospective observational economic study	∼200 elderly psychiatric patients (≥65 years), combinatorial PGx testing integrated into routine therapeutic management	Healthcare utilization and costs, including hospitalizations, emergency visits, and total medical costs	PGx-tested patients showed reduced healthcare utilization, including fewer hospitalizations and emergency visits, and lower overall medical costs during follow-up compared with controls	Combinatorial PGx testing in elderly psychiatric patients was associated with economic benefits and reduced healthcare utilization, supporting its potential value in geriatric psychiatric care
102	Brown et al. (2017)	USA	Primary care; inpatient/outpatient NR	Retrospective observational economic study	∼500 patients in primary care, receiving mental health care for depression and anxiety; combinatorial PGx-testing (CYP2D6, CYP2C19, CYP1A2, CYP3A4/5 and selected PD genes)	Medication-related costs, pharmacy expenditures, and healthcare utilization	Patients receiving PGx-guided care had significantly lower medication costs and reduced use of medications predicted to have gene–drug interactions. Cost savings were primarily driven by improved medication selection and reduced trial-and-error prescribing.	Combinatorial PGx testing in primary care mental health settings was associated with meaningful medication cost savings, supporting its economic utility, although findings are observational and not causal.
104	Crutchley et al. (2022)	USA	Inpatient	Secondary (post hoc) analysis of an observational cohort	∼1,500 patients with MDD or depressive symptoms receiving AD treatment; PGx testing applied to existing medication regimens	Prevalence of actionable DGIs and changes in prescribing following PGx report availability	A high proportion of patients had at least one actionable drug–gene interaction. Prevalence and type of DGIs differed significantly across ancestry groups. PGx information identified opportunities to optimize AD selection and dosing, particularly in underrepresented populations.	DGI are common in depressed patients, and their prevalence varies by ancestry
105	Ruaño et al. (2021)	USA	Inpatient	Secondary (post hoc) analysis of an implementation trial	∼1,500 MDD patients in the parent CYP-GUIDES cohort; analyzed subset varies by availability of CYP2D6 and timeline data	Timing and operational impact of PGx result availability on clinical decision-making	CYP2D6 functional stratification identified meaningful variability in AD metabolism. Clinical utility of PGx results was greatest when reports were delivered early in the treatment timeline, before multiple medication failures occurred. Delayed PGx testing reduced potential clinical impact.	Early implementation of CYP2D6 PGx testing maximizes clinical usefulness. Operational timing is a critical determinant of PGx effectiveness in real-world psychiatric care.
106	Ramsey et al. (2021)	USA (VA medical centers)	Non-inpatient at enrollment	Cross-sectional/ baseline analysis within a pragmatic randomized trial (PRIME Care)	1944 patients with MDD, PGx testing for DGI	Prevalence of predicted AD gene–drug interactions	A substantial proportion of patients had at least one predicted DGI at baseline. Interactions were common across AD classes, particularly SSRIs and SNRIs metabolized by CYP2D6 and CYP2C19.	Predicted gene–drug interactions are highly prevalent among patients with MDD receiving ADs, supporting the clinical rationale for PGx testing prior to or early in treatment.

*Country/region* and *setting* are reported as explicitly stated in each source. When inpatient/outpatient status or recruitment setting was not specified in the original report, it is marked as NR (not reported). “Mixed” indicates that both inpatient and outpatient populations (or multiple clinical contexts) were explicitly included. Abbreviations: AD, antidepressant; ADR, adverse drug reactions; ASD, autism spectrum disorder; CDRS-R, Children’s Depression Rating Scale–Revised; DGI, drug–gene interaction; EMC, early medication change; FEP, first-episode psychosis; HAM-D/HAMD, hamilton depression rating scale; ICER, incremental cost-effectiveness ratio; MDD, major depressive disorder; NGF, nerve growth factor; NR, not reported; OL, open-label; PGx, pharmacogenetics/pharmacogenomics; PHQ-9, Patient Health Questionnaire-9; PK/PD, pharmacokinetic/pharmacodynamic; PRS, polygenic risk score; RCT, randomized controlled trial; SSRI, selective serotonin reuptake inhibitor; TAU, treatment as usual; TCA, tricyclic antidepressant; TDM, therapeutic drug monitoring; VA = U.S., veterans health administration.

**TABLE 2:** Reviews and meta-analyses referring to the role of PGx in psychopharmacology.

References	Authors	Methodology	Intervention	Outcomes	Results	Conclusions
Antidepressants — PK/PD and biomarker evidence (reviews/meta-analyses)						
1	Fornaguera et al. (2025)	SR (∼50 studies)	AD	Treatment response, remission, drug tolerability, and ADR	Evidence consistently supports CYP2D6 and CYP2C19 as predictors of AD exposure and safety. Evidence for predicting efficacy using PD genes remains inconsistent. Recent studies increasingly support combinatorial PGx approaches and clinical decision support systems rather than single-gene testing	PGx has clear clinical relevance for AD safety and dosing in MDD
2	Grant et al. (2025)	SR (∼100 studies)	AD	Treatment response, remission, drug tolerability, and ADR	The most consistent and clinically actionable evidence supports PK genes (CYP2D6, CYP2C19) for predicting AD exposure and safety. Evidence for PD genes and polygenic models remains heterogeneous with limited replication	AD PGx in MDD is supported by robust evidence for CYP-based metabolism effects, while broader genetic prediction of efficacy remains investigational
3	Fabbri and Serretti (2020)	NR	AD	Standardized definitions of response, remission, tolerability, and ADR, with sufficient follow-up duration	Single-gene approaches are likely insufficient; multigene and combinatorial models may better capture AD response variability	Future AD PGx studies must adopt rigorous, standardized, and clinically grounded designs to translate genetic findings into reliable clinical tools
8	Correia et al. (2022)	SR (42 studies)	AD	Treatment effectiveness (response, remission) and safety/tolerability (ADR, discontinuation)	The strongest and most consistent evidence supported CYP2D6 and CYP2C19 as predictors of AD exposure, tolerability, and risk of adverse events. Evidence for PD biomarkers was inconsistent and often non-replicated	PGx testing may improve AD safety and dose optimization, particularly for CYP-metabolized drugs, but evidence for predicting efficacy remains limited
33	Chang et al. (2018)	NR with applied clinical guidance	PGx-guided AD prescribing using existing guidelines and decision support tools	Medication safety, dose optimization, reduction of adverse drug reactions, and improved tolerability	Age-related PK changes, polypharmacy, and comorbidity increase the clinical relevance of PGx in late-life depression. PGx is most useful for preventing adverse effects and guiding dosing rather than predicting AD efficacy	PGx guidelines and decision-support tools can meaningfully support safer AD use in late-life depression, particularly for CYP-metabolized drugs
34	Stingl et al. (2015)	NR	Metabolic mechanisms and clinical implications	Impact of genetic polymorphisms on psychotropic drug metabolism	CYP2D6 and CYP2C19 polymorphisms explain substantial interindividual variability in psychotropic drug response and tolerability; dosing adjustments or alternative drug choices may be necessary	CYP2D6 and CYP2C19 polymorphisms are central determinants of psychotropic drug metabolism
39	Stein et al. (2021)	SR (82 studies) and MTA (16 studies)	ADs	5-HTTLPR polymorphisms in relation to response and tolerability of AD	Short (S) allele carriers showed an increased risk of antidepressant discontinuation and adverse effects, particularly in European ancestry populations	The 5-HTTLPR polymorphism may serve as a marker for AD outcomes in psychiatric disorders among individuals of European descent
42	Tang et al. (2020)	SR (16 studies) and MTA (11 studies)	ADs	Association between COMT Val108/158 Met and AD response	COMT rs4680 variants showed significant associations with AD treatment, but the results were highly dependent on the individual study. Patients with the GG or AG + GG genotype, in comparison to AA, had a better response to ECT treatment	The COMT Val108/158Met polymorphism is not a reliable universal predictor of antidepressant treatment response, though it may be associated with response in Asian populations
45	Colle et al. (2015)	NR (5 GWAS and 30 association studies)	ADs	AD efficacy in relation to BDNF/TRKB/P75NTR polymorphisms	The only SNP that benefits from at least three positive studies is the BDNF Val66Met polymorphism (rs6265)	The clinical utility of BDNF in treatment selection is unclear
48	Kim et al. (2021)	SR (31 studies)	ADs	RNA expression changes during AD treatment	Multiple RNAs (including immune-, neuroplasticity-, and stress-related transcripts) showed treatment-associated expression changes, but findings were heterogeneous and not consistently replicated	Heterogeneous findings, no clinically validated RNA biomarkers have been established
9	Santoro et al. (2016)	NR	Genetic risk review	Contribution of common variants to disease risk, shared genetic architecture, and pleiotropy across psychiatric disorders	Common variants individually have small effect sizes but collectively contribute to psychiatric disorder risk. Substantial genetic overlap exists across disorders, challenging strict diagnostic boundaries	Psychiatric disorders are polygenic, with many common variants contributing small effects
Antidepressants — PGx-guided RCTs and pragmatic trials						
12	Brown et al. (2020)	SR and MTA (4 prospective clinical trials)	Combinatorial PGx-guided AD treatment	Response and remission rates in MDD	MTA showed significantly higher response and remission rates in PGx-guided care compared with TAU. Effect sizes were modest but consistent across trials, with the greatest benefit in patients whose baseline medications had predicted DGI.	Combinatorial PGx testing demonstrates statistically significant clinical utility for improving AD response and remission
13	Skryabin et al. (2023)	SR and MTA (6 clinical trials)	AD	Response and remission rates in MDD	MTA demonstrated significantly higher response and remission rates in patients managed with PGx-based CDSS compared with TAU. Effect sizes were small to moderate but consistent across studies	PGx CDSS provide measurable clinical benefit in the treatment of MDD, supporting their use as an adjunct to standard clinical decision-making
14	Wang et al. (2023)	SR and MTA (7 RCTs)	ADs	Response and remission rates in MDD	PGx-guided treatment significantly improved response and remission rates compared with TAU. Effects on symptom score reduction were modest. No consistent reduction in ADR was demonstrated across studies	PGx-guided treatment provides statistically significant but modest clinical benefit for patients with MDD, supporting its use as an adjunct to standard care
15	Cheng et al. (2023)	SR and MTA (6 RCTs)	AD	Response and remission rates in TRD	PGx-guided treatment significantly ↑ response and remission rates compared with TAU in TRD. Effect sizes were small to moderate and consistent across studies	PGx-guided treatment provides clinically meaningful benefits for patients with TRD, supporting targeted PGx implementation in this high-need population
16	Zhang et al. (2025)	SR and MTA, cumulative MTA (10 RCTs)	ADs	Response and remission in adults with MDD	PGx-guided treatment significantly improved response and remission compared with TAU. Benefits were more pronounced in patients with baseline actionable gene–drug interactions and in treatment-resistant subgroups. Cumulative MTA showed increasing precision and stability of effect estimates over time	PGx-guided AD treatment provides robust and increasingly consistent clinical benefit in MDD, particularly in patients with prior non-response or actionable PGx findings
Antipsychotics and mood stabilizers — clinical evidence						
55	Zhang et al. (2020)	SR and MTA (15 studies)	Oral risperidone	PK parameters of risperidone and 9-hydroxy risperidone	CYP2D6 IM had risperidone Css 2.35-fold higher than NM. PM had a 6.20-fold higher risperidone dose-adjusted concentration than NM. IM had also a higher Css vs. NM for the active moiety	Impaired CYP2D6 activity (IM, PM) is robustly associated with ↑ exposure to risperidone and its active moiety in adults
56	Dodsworth et al. (2018)	SR (10 studies)	Risperidone	Risperidone plasma concentration/PK, treatment efficacy, ADR in children and adolescents	IM and PM presented ↑risperidone plasma concentrations vs. NM. Hyperprolactinemia and EPS were the most frequently examined adverse effects, with mixed findings across studies	CYP2D6 phenotype has a clear and consistent effect on risperidone PK in children and adolescents, but evidence linking genotype to clinical outcomes (efficacy or adverse effects) remains insufficient
57	Maruf et al. (2021)	SR (n = 12 studies)	Antipsychotics	PK and drug exposure, treatment efficacy and adverse events in the pediatric population	Consistent evidence that CYP2D6 IM and PM have higher antipsychotic plasma concentrations, particularly for risperidone. Some studies suggested ↑ risk of hyperprolactinemia or EPS in PM, but findings were not uniform	CYP2D6 phenotype has a clear impact on antipsychotic PK in children and youth, but current evidence is insufficient to draw firm conclusions about clinical outcomes
58	Na Takuathung et al. (2019)	SR and MTA (14 studies)	CYP1A2-metabolized antipsychotics, primarily clozapine and olanzapine (with some studies including other substrates)	PK parameters	CYP1A2*1F A allele was associated with significantly lower clozapine and olanzapine plasma concentrations compared with C/C genotype carriers. MTA showed a significant reduction in dose-adjusted clozapine concentrations in A-allele carriers	CYP1A2 genetic polymorphisms, especially 1F, significantly influence antipsychotic PK, particularly for clozapine and olanzapine. Environmental factors (notably smoking) have a major modifying effect and must be considered alongside genotype when interpreting CYP1A2 effects
62	Mao et al. (2023)	SR (13 studies)	Olanzapine	PK/PD in pediatric and adult populations	Apparent clearance and other PK parameters were related to gender and smoking status	A higher dosage of olanzapine may be required for men or heavy smokers than for women or nonsmokers to reach the same exposure
63	Aldaz et al. (2021)	SR (27 studies)	ADs, antipsychotics	PK, PGx	Six studies on ADs found PGx-based dosing improved efficacy. Only one study on antipsychotics found fewer ADR with PGx-guided dosing in patients with CYP2D6 metabolized drugs	The evidence available on PK and PD-based personalization of treatment with psychoactive drugs is scarce
64	de Leon (2020)	NR	Risperidone, paliperidone, clozapine	CYP2D6 (risperidone, paliperidone) and CYP1A2 (clozapine) metabolizer status, plus therapeutic drug monitoring (plasma concentrations)	CYP2D6 PM, UM show clinically meaningful differences in exposure to risperidone and paliperidone. CYP1A2 activity, heavily influenced by smoking and inflammation, is the dominant determinant of clozapine levels	PGx alone is insufficient for dose individualization; TDM is essential, particularly for clozapine
66	Ma et al. (2019)	SR and MTA (16 studies)	Risperidone	Treatment response in relation to DRD2 and DRD3 gene polymorphisms	DRD2 Taq1A A1 allele carriers showed better treatment response to risperidone compared with A2/A2 genotype. DRD2 −141C Ins/Del polymorphism showed no consistent association with risperidone efficacy. DRD3 Ser9Gly polymorphism was not significantly associated with treatment response in pooled analyses	Certain DRD2 polymorphisms, particularly Taq1A, are associated with risperidone treatment response, whereas evidence for DRD3 variants is limited
67	Huang et al. (2016)	SR and MTA (12 studies)	Antipsychotics	Clinical response to antipsychotic treatment in relation to the COMT Val158Met (rs4680) polymorphism	Overall, the MTA showed no significant association between the COMT Val158Met genotype and antipsychotic response across all patients	COMT Val158Met alone is not a strong or reliable predictor of antipsychotic treatment response in schizophrenia or schizoaffective disorder
68	Ma et al. (2021)	SR and MTA (20 studies)	Antipsychotics	Clinical efficacy of antipsychotic treatment in relation to the COMT Val158Met (rs4680) polymorphism	In the overall pooled analysis, the COMT Val158Met polymorphism showed a small but statistically significant association with antipsychotic efficacy. Met allele carriers demonstrated greater symptom improvement compared with Val/Val homozygotes	The COMT Val158Met polymorphism may modestly influence antipsychotic treatment response, particularly in East Asian populations, but the effect size is small and insufficient for standalone clinical decision-making
69	Calabrò et al. (2018)	NR	Antipsychotics	Clinical response to antipsychotics in relation to various candidate genes	Variants in dopaminergic (DRD2/DRD3) and serotonergic (HTR2A/HTR2C) genes show the most consistent, though modest, associations with treatment response. COMT and BDNF variants may influence cognition and symptom dimensions rather than global response. Most reported effects are small, heterogeneous, and population-dependent	Genetic variation within molecular targets of antipsychotics may contribute to interindividual variability in treatment response and symptom profiles, but findings are inconsistent and insufficient for clinical implementation
70	Teng et al. (2023)	SR and MTA (43 studies)	Antipsychotics	Treatment response, adverse drug reactions, and PK variability in relation to various candidate genes	Strongest and most consistent evidence was for CYP2D6–antipsychotic exposure relationships, particularly for risperidone and aripiprazole. PD gene associations (e.g., COMT, DRD2) showed small, inconsistent, and population-specific effects	The current evidence does not yet support widespread precision prescribing in schizophrenia
71	Zhang et al. (2016)	SR and MTA (46 studies)	Antipsychotics, mainly SGA, that are associated with weight gain	Antipsychotic-induced weight gain in relation to various candidate genes	HTR2C −759C/T (rs3813929) showed the most consistent association, with the T allele protective against weight gain (pooled effect significant). MC4R variants were associated with ↑ weight gain risk, particularly with clozapine and olanzapine. Many other candidate gene associations showed inconsistent or null results after MTA.	Overall effect sizes are modest, and genetic testing alone is insufficient to predict weight gain risk without considering clinical factors
73	Yoshida et al. (2020)	NR	Antipsychotics	PK, treatment response, and adverse events in relation to various candidate genes	Strongest and most clinically actionable evidence is for CYP2D6 (risperidone, aripiprazole) and CYP1A2 (clozapine). PD gene associations (e.g., DRD2, COMT) show small, inconsistent effects. PGx should complement, but not replace, the TDM.	PGx testing has clear value for antipsychotic metabolism genes, while receptor-level genetics remain research tools rather than clinical decision aids
74	Miura et al. (2016)	SR and MTA (10 studies)	Antipsychotics	Antipsychotic-induced prolactin levels in relation to DRD2 locus variants	DRD2 Taq1A A1 allele carriers had significantly higher prolactin levels compared with A2/A2 genotype carriers (pooled standardized mean difference statistically significant). −141C Ins/Del polymorphism showed no consistent association with prolactin levels	Genetic variation at the DRD2 locus, particularly Taq1A, contributes to interindividual variability in antipsychotic-induced hyperprolactinemia
75	Calafato et al. (2020)	SR and MTA (11 studies)	Antipsychotics	Antipsychotic-induced prolactin levels in relation to CYP2D6 metabolizer phenotypes	CYP2D6 PMs had significantly higher prolactin levels compared with normal metabolizers (pooled effect significant). IMs showed an intermediate increase in prolactin levels. Associations were strongest for risperidone/paliperidone, consistent with CYP2D6-dependent metabolism. Moderate heterogeneity was observed across studies	Reduced CYP2D6 activity is associated with ↑ the risk of antipsychotic-induced hyperprolactinaemia
76	Nasyrova et al. (2023)	SR (25 studies)	Antipsychotics (typical and atypical)	Antipsychotic-induced akathisia in relation to a broad candidate gene set	Most frequently reported associations involved dopaminergic (DRD2/DRD3) and serotonergic (HTR2A/HTR2C) genes. CYP2D6 variants were linked to akathisia risk in some studies, particularly with CYP2D6-metabolized antipsychotics. Results were inconsistent, with many findings not replicated across studies	While multiple SNPs have been proposed as biomarkers for antipsychotic-induced akathisia, current evidence is heterogeneous and insufficient for clinical application
77	Pagani et al. (2019)	SR (41 studies)	Lithium	Lithium treatment response in relation to a broad range of candidate genes	Most candidate gene associations showed inconsistent or non-replicated results. Genome-wide studies suggested several novel loci, but findings lacked replication and clinical validation. No single genetic marker demonstrated robust predictive power across studies	Despite extensive research, no PGx marker has sufficient evidence for routine clinical prediction of lithium response
78	Yoshida et al. (2021)	SR (34 studies)	Antipsychotics and other psychotropics	Treatment response, adverse drug reactions, and drug tolerability in ID and ASD populations in relation to genes involved in PK and PD	Evidence was sparse and heterogeneous. CYP-based metabolism effects showed the most consistent signals, particularly for antipsychotic exposure and ADR. PD gene associations were inconsistent and rarely replicated	Current PGx evidence in ID and ASD is limited and insufficient for routine clinical use
Implementation, economics and real-world context						
17	Bousman et al. (2016)	Personal view/narrative evaluation (literature + internet search; adapted UKGTN-expanded ACCE framework)	Commercial pharmacogenetic-based decision-support tools in psychiatry (multi-gene PGx panels for antidepressants/antipsychotics)	Tool evaluation domains: Analytical validity, clinical validity (evidence level for included genes), clinical usefulness/utility (efficacy/effectiveness evidence), feasibility/affordability, ethical/legal/social implications	Identified ∼22 commercial tools across >20 countries; panels mainly PK-focused (CYP2D6/CYP2C19 in all tools). Public data on assay performance/validity often unavailable. Many genes included had low or preliminary evidence by PharmGKB-level appraisal; clinical utility evidence was limited and concentrated in a few tools (e.g., GeneSight, CNSDose, Genecept), with concerns about conflicts of interest and lack of independent evaluation	Commercial PGx decision-support tools are widespread, but evidence for routine adoption remains limited; CYP2D6/CYP2C19 are the most defensible components, and independent validation plus standardization are needed before broad implementation
18	Bousman et al. (2017)	SR/comparative review (6 commercial PGx testing platforms)	Commercial PGx testing for CYP2D6 and CYP2C19	Number of alleles tested, predicted metabolizer phenotypes, reporting transparency, and clinical interpretability	Substantial variability in allele coverage across companies, particularly for reduced-function and rare alleles. Differences in phenotype assignment and reporting language were observed, leading to potential discrepancies in clinical interpretation	Commercial PGx tests differ meaningfully in CYP2D6 and CYP2C19 allele coverage and reporting practices, which may affect clinical decision-making in psychiatry
19	Health quality Ontario (2017)	SR and HTA	Assurex GeneSight psychotropic PGx test	Depression symptom improvement, response, remission	Some studies suggested greater symptom improvement with GeneSight-guided care, but results were inconsistent and often based on *post hoc* or secondary analyses	At the time of review, evidence was insufficient to support routine public reimbursement of GeneSight testing for psychotropic medication selection
20	Morris et al. (2022)	SR (77 economic evaluations)	PGx testing to guide prescribing/dosing according to CPIC recommendations	Cost-effectiveness, cost–utility (e.g., cost per QALY), and cost savings	Most studies reported PGx testing to be cost-effective or cost-saving, particularly when testing was pre-emptive or applied to drugs with a high risk of serious adverse events. Evidence was strongest for oncology and cardiology, with growing but more variable evidence in psychiatry	PGx testing for drugs with CPIC guidelines is often cost-effective, supporting broader implementation, though results depend on test costs, population risk, and healthcare system context
21	Koopmans et al. (2021)	SR and MTA (>400 population-based studies)	Population-level genetic frequency analysis	Probability estimates of metabolizer phenotypes (poor, intermediate, normal, ultrarapid) by geographic region and ancestry	There is substantial global variability in CYP2D6 and CYP2C19 metabolizer frequencies. PM and UM prevalences differ markedly across regions, with important implications for drug response and ADR	Worldwide variation in CYP2D6 and CYP2C19 is pronounced, underscoring the need for ancestry-aware PGx in clinical practice and guideline development
22	Wu et al. (2025)	Scoping review (>100 implementation studies)	Implementation of PGx testing in hospital clinical workflows	Implementation strategies, adoption, feasibility, fidelity, acceptability, and sustainability	Successful hospital implementation relies on clinical decision support integration, multidisciplinary governance, education, and pre-emptive testing models. Evidence for clinical benefit is strongest when PGx results are embedded in EHRs with actionable alerts	Effective implementation of PGx testing in hospitals requires system-level strategies rather than standalone testing
86	Thomas (2020)	NR	Use of PGx information, drug–drug interaction management, and DDGI awareness to optimize prescribing	Reduction of ADR, improved drug safety, optimized dosing in older adults	CYP450 enzyme activity varies substantially by ancestry, age, and co-medication use. Seniors are at particularly high risk for DDI and DDGI due to polypharmacy. Integrating PGx with medication review can substantially reduce ADE risk	Personalized prescribing for seniors should integrate PGx, ancestry-informed metabolism differences, and careful management of DDIs/DDGIs to reduce adverse drug events and improve safety
107	Ranasinghe et al. (2024)	SR (80 studies)	N/A	Allele frequencies, genotype–phenotype relationships, drug response, adverse drug reactions, and clinical relevance	Significant differences in allele frequencies were observed between Sri Lankan and other global populations, particularly for CYP and HLA genes. Psychiatric PGx evidence remains limited, while stronger data exist for oncology and infectious diseases. Clinical implementation is constrained by limited infrastructure and a lack of national guidelines	Highlights the need for population-specific PGx data, local guidelines, and capacity building before routine clinical implementation
108	Di Nunno et al. (2021)	NR	N/A	Interpretation of drug concentrations, cause of death, toxicity, and interindividual variability in forensic investigations	PGx variability can explain unexpected drug concentrations and toxicological findings. Incorporating PGx may improve accuracy in forensic case interpretation, including differentiation between overdose, adverse drug reactions, and therapeutic misadventure	Integrating PGx into forensic toxicology supports a multidisciplinary approach that can improve the interpretation of toxicological evidence

Abbreviations: AD, antidepressant; ADR, adverse drug reactions; ADE, adverse drug events; ASD, autism spectrum disorder; BDNF, Brain-Derived Neurotrophic Factor; CDSS, clinical decision support system; COMT, Catechol-O-Methyltransferase; CPIC, clinical pharmacogenetics implementation consortium; DDGI, Drug-Drug-Gene Interaction; DDI, Drug-Drug Interactions; DGI, Drug-Gene Interactions; EPS, extrapyramidal symptoms; GWAS, Genome-Wide Association Study; HLA, human leukocyte antigen; ID, intellectual disability; IM, intermediate metabolizer; MC4R = Melanocortin-4, receptor; MDD, major depressive disorder; MTA, meta-analysis; N/A = not applicable; NM, normal metabolizer(s); NR, narrative review; PD, pharmacodynamic; PM, poor metabolizer; PGx, pharmacogenetics/pharmacogenomics; QALY, Quality-Adjusted Life Year; RCT, randomized clinical trial; SNRI, serotonin and norepinephrine reuptake inhibitor; SR, systematic review; SSRI, selective serotonin reuptake inhibitor; TDM, therapeutic drug monitoring; TRD, treatment-resistant MDD; TAU, treatment as usual; UM, ultrarapid metabolizer(s).

**TABLE 3:** Consensus papers, guidelines and other sources referring to the role of PGx in psychopharmacology.

References	Authors	Methodology	Intervention	Outcomes	Results	Conclusions
10	Bousman et al. (2021)	Consensus paper	PGx testing for guiding psychotropic medication selection and dosing	Medication safety, tolerability, dose optimization, ADR, and treatment response	Strong evidence supports CYP2D6 and CYP2C19 testing for improving safety and dosing. Evidence for PD genes and combinatorial panels is mixed and condition-specific. PGx testing should complement, not replace, clinical judgment and therapeutic drug monitoring	PGx testing has clear clinical validity for CYP2D6 and CYP2C19 in psychiatry
11	Beunk et al. (2024)	Clinical practice guideline (DPWG)	Antipsychotics	PGx-guided prescribing and dosing of antipsychotics in relation to CYP2D6, CYP3A4, and CYP1A2 variants	CYP2D6: Dose reduction or alternative antipsychotics recommended for poor and IM when prescribing CYP2D6-dependent drugs (e.g., risperidone, aripiprazole). CYP1A2: Dose adjustments are recommended for drugs such as clozapine and olanzapine, particularly in poor metabolizers and in the context of smoking status. CYP3A4: Consideration of altered exposure, though evidence is weaker than for CYP2D6 and CYP1A2	The DPWG guideline provides actionable, gene-specific dosing recommendations for antipsychotics, supporting routine clinical use of CYP2D6 and CYP1A2 PGx information, with cautious interpretation for CYP3A4
23	Ginsburg et al. (2021)	Pragmatic trials network description/methods paper	Implementation of genomic and PGx testing within pragmatic clinical trials	Clinical utility, implementation, effectiveness, and scalability of genomic medicine in real-world healthcare settings	The IGNITE PTN provides a coordinated infrastructure to generate high-quality, real-world evidence on the value of genomics in routine care	Pragmatic, multisite trials embedded in healthcare systems are essential for demonstrating the real-world value of PGx and other genomic applications
24	Cavallari et al. (2017)	Institutional profile/program description	Pre-emptive and reactive PGx testing integrated into routine clinical care	Program reach, number of tests ordered, CDS alert usage, and examples of clinical impact (no formal efficacy analysis)	Demonstrates feasibility of system-level PGx implementation, emphasizing pharmacist-led services and EHR integration	The UF health personalized medicine program provides a scalable model for real-world PGx implementation within an academic health system
25	Maruf et al. (2020)	Policy and practice overview	PGx testing to inform psychotropic medication selection and dosing	Clinical validity, clinical utility, availability, and interpretability of PGx tests	Evidence is strongest for CYP2D6 and CYP2C19 in predicting drug metabolism and ADR. Considerable variability exists across commercial tests in gene coverage, reporting style, and clinical recommendations	PGx testing relevant to psychiatry is available in Canada, but standardization, education, and clearer reimbursement pathways are needed to support consistent and evidence-based clinical implementation
26	Bhimpuria et al. (2024)	QI project	PGx testing integrated into primary care prescribing (CYP2D6, CYP2C19)	Prescribing changes, identification of gene–drug interactions, and clinical decision support utilization	PGx testing was feasible in routine primary care and led to frequent medication changes based on identified gene–drug interactions. Clinician confidence in prescribing ↑, and PGx results informed long-term medication management	PGx testing can be successfully implemented in primary care as part of a QI initiative, supporting safer and more personalized prescribing, although controlled studies are needed to quantify clinical benefit
32	Fabbri et al. (2017)	Expert consensus (WFSBP)	Evidence synthesis (CYP2D6, CYP2C19, SLC6A4, HTR2A, BDNF, COMT, inflammatory and neuroplasticity-related genes)	Risk of MDD, AD response, remission, and tolerability	PGx markers affecting drug metabolism are currently the most reliable for clinical use. Most genetic, epigenetic, and transcriptomic markers of AD response remain research tools rather than ready for routine practice. Consider CYP-based PGxs to improve safety and dosing; do not use single PD or epigenetic markers alone to guide AD selection	While genetics, epigenetics, and gene expression contribute to MDD and AD response, only PK PGx currently meets criteria for cautious clinical application; broader precision psychiatry approaches require further validation
107	Bousman et al. (2023)/CPIC	Clinical practice guideline (CPIC)	SSRI/SNRI antidepressants	PGx-guided dosing recommendations; dose optimization; ADR risk mitigation	Provides genotype/phenotype-informed dosing recommendations for selected SSRIs/SNRIs, including CYP2C19-related guidance for citalopram/escitalopram and broader CYP2D6/CYP2C19 considerations across multiple agents	Actionable, gene–drug-specific dosing guidance to support implementation and CDS integration; complements consensus statements and jurisdiction-specific guideline bodies

Abbreviations: AD, antidepressant; ADR, adverse drug reactions; CDS, clinical decision support; EHR, electronic health record; DPWG, dutch pharmacogenetics working group; MDD, major depressive disorder; PD, pharmacodynamics; PGx, pharmacogenetics/pharmacogenomics; QI, quality improvement; WFSBP, world federation of societies of biological psychiatry; CPIC, clinical pharmacogenetics implementation consortium.

**TABLE 4:** Additional studies reviewed and summarized separately.

References	Authors	Methodology	Participants and intervention	Outcomes	Results	Conclusions
37	Huang et al. (2021)	Prospective PK study	90 Chinese healthy subjects receiving citalopram	The effect of CYP2C19 genotype on escitalopram PK	Genotype-guided dosing improved tolerability; PMs had higher exposure, ↑ Css vs. EM and IM	Genetic testing before medication initiation in PMs should be considered in Chinese individuals
40	Outhred et al. (2016)	Neuroimaging genetic study	36 Caucasian healthy females receiving a single dose of escitalopram vs. placebo	fMRI response of the left amygdala and right inferior frontal gyrus in relation to 5-HTTLPR polymorphisms	5-HTTLPR modulates SSRI effects. Escitalopram may enhance the negative functional connectivity in homozygous L allele participants and least in S homozygous participants	AD treatment produces differential treatment outcomes dependent on the 5-HTTLPR polymorphism
50	Fageera et al. (2021)	DB, PC, cross-over pediatric RCT with epigenetic analysis	230 children with ADHD, methylphenidate treatment	Symptom change under active treatment vs. placebo, association of COMT methylation with treatment response	Specific COMT genotypes were significantly associated with higher methylation at a key CpG site. Methylation at the same site negatively correlated with placebo response and showed a trend with methylphenidate response	Preliminary evidence that COMT genetic variation influences DNA methylation, and COMT methylation at specific CpG sites may be involved in modulating ADHD treatment responses to methylphenidate and placebo
59	Cabaleiro et al. (2015)	Prospective, OL, PGx study	30 healthy Caucasian volunteers, treated with a single dose of quetiapine	PK parameters, PD (sedation, cardiovascular effects), adverse events	Quetiapine AUC was associated with CYP1A2 polymorphisms and clearance with DRD3; prolactin iCmax was higher in carriers of CYP2C19 and AGT variants; somnolence was associated with CYP1A1 and CYP2C9 polymorphisms	Genetic variability in CYP1A2, DRD3, CYP2C19, AGT, CYP1A1, and CYP2C9 may influence quetiapine PK, PD, and tolerability in healthy volunteers
60	Zubiaur et al. (2021)	Prospective, OL, PGx study	49 healthy Caucasian volunteers, quetiapine exposure	PK parameters and COMT, CYP3A5, CYP2B6, ABCG2 polymorphisms	CYP3A5*1 carriers (expressers) showed significantly lower quetiapine exposure, and CYP2B6 variants were associated with differences in quetiapine clearance and exposure. ABCG2 polymorphisms influenced quetiapine plasma concentrations, consistent with altered drug transport. COMT variants were associated with PK variability, possibly reflecting indirect neurochemical or regulatory effects	Genetic variation in CYP3A5, CYP2B6, ABCG2, and COMT contributes to interindividual variability in quetiapine PK
61	Koller et al. (2020)	Randomized, OL, multiple-dose crossover PGx trial	24 healthy Caucasian volunteers, aripiprazole or olanzapine were orally administered	Pupillary reflex in relation to polymorphisms in CYP3A, HTR2A, UGT1A1, DRD2 and ABCB1	Polymorphisms in CYP3A, HTR2A, UGT1A1, DRD2 and ABCB1 affected pupil size, the time of onset of constriction, pupil recovery and constriction velocity. Aripiprazole, dehydro-aripiprazole, and olanzapine PK were significantly affected by polymorphisms in CYP2D6, CYP3A, CYP1A2, ABCB1, and UGT1A1 genes	Aripiprazole and its main metabolite, dehydro-aripiprazole, altered pupil contraction, but olanzapine did not have such an effect
96	Strawn et al. (2021)	RCT, protocol paper	∼200 children and adolescents, PGx-guided treatment with escitalopram vs. standard dosing	Change in anxiety symptom severity in relation to CYP2C19 genotypes, treatment response and tolerability	Not yet available	This paper outlines the rationale, design, and methods for a rigorously blinded pediatric PGx trial
103	Ghanbarian et al. (2024)	Study protocol for a health economic simulation model	Adults with MDD, PGx-guided therapy, and other pharmacological treatment strategies vs. standard care	Cost-effectiveness outcomes, including costs, QALYs, and ICERs	Not yet reported	This paper describes the design and planned methods of a simulation model; no clinical or economic results are reported
109	Smith et al. (2019)	Pragmatic clinical trial	235 patients with chronic pain vs. 135 in usual care, CYP2D6-guided opioid prescribing	Pain control, measured by validated pain intensity and interference scales	Among CYP2D6 intermediate and poor metabolizers, PGx-guided opioid therapy resulted in significantly improved pain control compared with usual care. Normal and ultrarapid metabolizers showed no significant benefit from PGx guidance	CYP2D6-guided opioid therapy improves pain outcomes in patients with reduced CYP2D6 activity, supporting targeted PGx implementation rather than universal testing

Abbreviations: AD, antidepressant; BD, double-blind; CYP, cytochrome P450; fMRI, functional magnetic resonance imaging; ICER, incremental cost-effectiveness ratio; MDD, major depressive disorder; OL, open-label; PC, placebo-controlled; PGx, pharmacogenetics/pharmacogenomics; PK, pharmacokinetics; PM/IM/EM/UM, poor/intermediate/extensive/ultrarapid metabolizer; QALY, quality-adjusted life year; RCT, randomized controlled trial; SSRI, selective serotonin reuptake inhibitor.

Studies are listed separately when they primarily report (i) healthy-volunteer PK/experimental findings, (ii) proxy outcomes (e.g., neuroimaging/physiological markers) rather than clinical symptom endpoints, (iii) protocol-only or modeling-only reports without outcome data in that publication, or (iv) indications outside the main psychopharmacology prescribing context.

### 3.1 Genetic evidence base for psychiatric pharmacogenomics

#### 3.1.1 Overall architecture of genetic influences on treatment response

Across psychiatric diagnoses, genetic influences on pharmacological response and tolerability are consistently polygenic and modest in effect size. Umbrella reviews and consensus papers show that most single variants explain only a very small proportion of the variance in treatment outcomes, with limited replication across cohorts and ancestries (Fornaguera and Miarons, 2025; Grant et al., 2025; Fabbri and Serretti, 2020; Santoro et al., 2016). Large-scale overviews of genomic contributions to psychiatric disorders highlight a similar pattern in disease risk: hundreds of common variants with small effect sizes distributed across many loci rather than a few major genes (Santoro et al., 2016). Within this landscape, PK genes, particularly CYP2D6 and CYP2C19, emerge as the most significant and clinically actionable markers across psychotropic classes, whereas PD markers (serotonergic, dopaminergic, neurotrophic, and catecholaminergic genes) show inconsistent associations with treatment response or adverse effects (Fornaguera and Miarons, 2025; Grant et al., 2025; Fabbri and Serretti, 2020; Santoro et al., 2016; Beunk et al., 2024; Fabbri et al., 2017; Chang et al., 2018).

#### 3.1.2 Antidepressant pharmacogenetics in major depressive disorder

##### 3.1.2.1 Pharmacokinetic markers

Multiple systematic reviews and consensus statements converge on CYP2D6 and CYP2C19 as the central PK determinants of antidepressant exposure and, indirectly, of response and tolerability (Fornaguera and Miarons, 2025; Grant et al., 2025; Fabbri and Serretti, 2020; Correia et al., 2022; Santoro et al., 2016; Beunk et al., 2024; Wu et al., 2025; Maruf et al., 2020; Fabbri et al., 2017; Chang et al., 2018). Early mechanistic reviews of psychotropic metabolism established that selective SSRIs, serotonin–noradrenaline reuptake inhibitors (SNRIs), and TCAs vary in their dependence on these enzymes, with poor metabolisers showing higher plasma concentrations and an increased risk of adverse events, and ultrarapid metabolisers showing subtherapeutic exposure (Stingl and Viviani, 2015). A recent comprehensive systematic review of antidepressant pharmacogenetics in MDD, covering 2019–2024, synthesised 29 studies (≈40,000 patients) and found that CYP2D6 and CYP2C19 phenotypes are consistently associated with antidepressant plasma levels and adverse events, and more variably with clinical response (Fornaguera and Miarons, 2025). Similar conclusions are drawn by an independent large-scale review of antidepressant pharmacogenetics (Grant et al., 2025) and by a systematic review focused on pharmacogenomic biomarkers of antidepressant efficacy and safety (Correia et al., 2022). Drug-specific evidence reinforces this pattern. Systematic reviews and meta-analyses show that CYP2D6 genotype strongly influences desipramine, nortriptyline, and venlafaxine exposure and toxicity (Stingl and Viviani, 2015; Berm et al., 2016) and that CYP2C19 variation is linked to escitalopram and citalopram tolerability and dose requirements in both adults and youth (Aldrich et al., 2019; Huang et al., 2021). Observational studies report similar findings for sertraline and other SSRIs (Yuce-Artun et al., 2016). Taken together, the genetic evidence supports a biologically coherent and reproducible PK signal for CYP2D6/CYP2C19–antidepressant pairs, particularly for TCAs and several SSRIs. However, effect sizes for clinical response and remission are modest, and many studies remain underpowered or limited to a single ancestry (Fornaguera and Miarons, 2025; Grant et al., 2025; Fabbri and Serretti, 2020; Correia et al., 2022; Santoro et al., 2016; Stingl and Viviani, 2015; Berm et al., 2016; Aldrich et al., 2019; Huang et al., 2021; Yuce-Artun et al., 2016).

##### 3.1.2.2 Pharmacodynamic markers

By contrast, pharmacodynamic candidate genes show far more inconsistent evidence. Meta-analyses of serotonin transporter (solute carrier family-6 member 4 (SLC6A4), 5-hydroxytryptamine transporter-linked polymorphic region (5-HTTLPR)) variation and antidepressant response or tolerability reveal statistically significant but small effects, often restricted to particular ancestries or to specific drug classes (Stein et al., 2021; Outhred et al., 2016). A systematic review and meta-analysis of serotonin transporter genetic variation concluded that, although some associations with response and adverse events are reproducible, the overall predictive value is low and not robust enough for routine clinical use (Stein et al., 2021). Similarly, serotonin receptor genes (e.g., HTR2A, HTR2C) and other serotonergic markers have yielded heterogeneous results. Individual studies and small meta-analyses have identified associations between HTR2C polymorphisms and SSRI response or side effects, but these are not consistently replicated across cohorts or drugs (Correia et al., 2022; Wang et al., 2023; Li et al., 2019).

For catecholaminergic genes, multiple meta-analyses address catechol-O-methyltransferase (COMT) Val158Met and related polymorphisms. A quantitative synthesis of COMT and antidepressant response in major depression found at most weak and inconsistent associations across studies (Tang et al., 2020; Yin et al., 2016). A broader review of catecholamine pathway polymorphisms (including COMT, SLC6A2, SLC6A3, and dopamine receptor genes- DRD2 and DRD4) similarly concluded that effect sizes are small and that results vary by ethnicity, outcome definition, and study design (Wang et al., 2023; Yin et al., 2015). Neurotrophic and other signalling genes have also been investigated. Variants in brain-derived neurotrophic factor (BDNF), tropomyosin receptor kinase B (TRKB), p75 neurotrophin receptor (p75NTR), nerve growth factor (NGF), and related pathways have been linked to antidepressant efficacy in single studies (Colle et al., 2015; Yeh et al., 2015). In addition, large candidate-gene analyses combining multiple markers and early clinical improvement suggest that genetic information may modestly improve prediction models (Kato et al., 2015). However, systematic reviews emphasise that no single PD marker has reached the level of evidence or reproducibility seen for CYP2D6/CYP2C19 (Fornaguera and Miarons, 2025; Grant et al., 2025; Fabbri and Serretti, 2020; Correia et al., 2022; Santoro et al., 2016; Beunk et al., 2024; Colle et al., 2015; Yeh et al., 2015; Kato et al., 2015).

In summary, PD markers contribute to a diffuse and fragile genetic signal, with many positive findings but limited replication and small incremental predictive value beyond clinical factors.

##### 3.1.2.3 Epigenetic and gene expression markers

Beyond DNA sequence variation, several reviews have examined epigenetic and gene expression markers as predictors of antidepressant response. A systematic review of RNA expression changes during antidepressant treatment identified multiple candidate transcripts and pathways, but highlighted substantial heterogeneity in methods, platforms, and clinical endpoints, with only a few signals replicated across studies (Kim et al., 2021). Epigenetic studies report associations between methylation of serotonin-related genes and treatment outcomes (Bruzzone et al., 2025). For example, methylation patterns in SLC6A4 and other serotonergic loci have been proposed as predictors of clinical improvement, while methylation of COMT has been associated with stimulant response in attention-deficit/hyperactivity disorder, suggesting potential relevance of epigenetic modulation in psychopharmacology more broadly (Fageera et al., 2021). Consensus position papers integrating genetic, epigenetic, and gene-expression findings emphasise that, while these markers deepen mechanistic understanding, their clinical utility remains exploratory and requires prospective validation in larger, standardised cohorts (Fabbri et al., 2017).

##### 3.1.2.4 Antidepressant pharmacogenetics in special populations

###### 3.1.2.4.1 Youth and paediatric populations

In children and adolescents, the evidence base is smaller but mirrors that of adults. Pharmacokinetic studies indicate that CYP2C19 metaboliser status influences escitalopram and citalopram exposure, adverse events, and, to a lesser extent, response, consistent with adult data (Huang et al., 2021; Poweleit et al., 2019). Similar observations have been made for sertraline metabolism in relation to CYP2B6 and CYP2C19 variation (Yuce-Artun et al., 2016). Clinical studies in paediatric anxiety and depressive disorders show that escitalopram pharmacokinetics and adverse events are modulated by CYP2C19 and CYP2D6 status, with preliminary data suggesting that metaboliser extremes may be at higher risk of side effects or suboptimal efficacy (Amitai et al., 2016). Additional work in youths at familial risk for bipolar disorder implicates CYP2C19 in mediating SSRI-related dysfunctional arousal, again via PK mechanisms rather than specific PD variants (Honeycutt et al., 2024). Evidence for PD markers in youth (e.g., SLC6A4, serotonergic receptors) is even more inconsistent than in adults, with small studies and no robust meta-analytic signal (Poweleit et al., 2019; Amitai et al., 2016).

Overall, in this particular population, PK genes appear relevant and largely consistent across the lifespan, while PD markers remain tentative.

##### 3.1.2.5 Late-life depression

In late-life depression, a systematic review of pharmacogenetic determinants of antidepressant therapy concluded that the most consistent findings again involve CYP2D6 and CYP2C19, affecting drug exposure and adverse events, whereas PD markers (serotonergic, catecholaminergic, neurotrophic) show sparse and non-replicated associations (Marshe et al., 2020). Application-focused reviews of pharmacogenetic guidelines and decision-support tools for depression in older adults emphasise the same hierarchy: PK markers with guideline-level evidence, PD markers as emerging but not yet practice-changing (Beunk et al., 2024; Marshe et al., 2020).

#### 3.1.3 Antipsychotic pharmacogenetics

##### 3.1.3.1 Pharmacokinetic markers

For antipsychotics, PK variation has been most extensively studied for CYP2D6, CYP1A2, CYP3A4, and CYP2B6. A systematic review and meta-analysis of CYP2D6 polymorphisms and risperidone pharmacokinetics demonstrated clear genotype-dependent differences in active moiety concentrations, supporting dose-adjustment strategies in poor and ultrarapid metabolisers (Zhang et al., 2020). Quantitatively, dose-adjusted risperidone concentrations were approximately 2.3-fold higher in intermediate metabolisers and more than six-fold higher in poor metabolisers compared with normal metabolisers, underscoring how much larger PK effects can be than typical symptom-level gains (Zhang et al., 2020). Similar conclusions arise from systematic reviews of risperidone treatment in children and adolescents and of CYP2D6 and antipsychotic outcomes in youth more broadly (Dodsworth et al., 2018; Maruf et al., 2021). A meta-analysis of CYP1A2 polymorphisms and antipsychotic pharmacokinetics confirmed that allelic variation contributes to inter-individual differences in clozapine and olanzapine exposure, but the effect size is modest and modulated by environmental inducers such as smoking (Na Takuathung et al., 2019).

Additional work on quetiapine, aripiprazole, and other second-generation antipsychotics indicates that variants in CYP2D6, CYP3A5, CYP2B6, and ABC transporters influence pharmacokinetics, though the evidence is primarily derived from small trials in healthy volunteers and population PK studies rather than from large clinical cohorts (Cabaleiro et al., 2015; Zubiaur et al., 2021; Koller et al., 2020; Mao et al., 2023).

Systematic reviews of combined pharmacokinetic and pharmacogenetic approaches to optimising psychiatric treatment conclude that PK-based dosing -especially when combined with therapeutic drug monitoring (TDM)- has the strongest empirical grounding in antipsychotic pharmacogenomics, whereas purely PD-based strategies are more speculative (Aldaz et al., 2021; de Leon, 2020).

##### 3.1.3.2 Pharmacodynamic markers of efficacy

Multiple meta-analyses have examined PD markers and antipsychotic response. A systematic review and meta-analysis of dopamine receptor polymorphisms found that variants in DRD2 are modestly associated with antipsychotic efficacy, particularly in first-episode psychosis, but with considerable heterogeneity and limited predictive value at the individual-patient level (Zhang et al., 2015; Ma et al., 2019). Meta-analyses of COMT Val158Met and antipsychotic response in schizophrenia and schizoaffective disorder show small and inconsistent effects, with some studies suggesting better cognitive response or symptom reduction in Met carriers, while others find no significant association (Huang et al., 2016; Ma et al., 2021). Similarly, a comprehensive review of genetic variants within molecular targets of antipsychotic treatment (dopaminergic, serotonergic, glutamatergic, and neuropeptide systems) concluded that no single PD marker currently justifies routine clinical testing for efficacy prediction (Calabrò et al., 2018). Umbrella reviews integrating multiple PD markers highlight the same pattern: numerous nominally positive associations, but few that survive rigorous correction for bias, heterogeneity, and multiple testing (Grant et al., 2025; Teng et al., 2023).

##### 3.1.3.3 Pharmacogenetic predictors of antipsychotic adverse effects

By contrast, the adverse effects of antipsychotics have yielded somewhat stronger genetic signals, although still insufficient for widespread clinical adoption. A large systematic review and meta-analysis of antipsychotic-induced weight gain identified multiple loci, including HTR2C, melanocortin-4 receptor (MC4R), BDNF, and others, associated with weight gain across several antipsychotics, but effect sizes were small and varied across drugs (Zhang et al., 2016). Focused meta-analyses confirm that HTR2C polymorphisms and variants in other regulators of fat-mass homeostasis contribute to weight gain in bipolar disorder and schizophrenia, yet the variance explained remains limited (Creta et al., 2015; Yoshida and Müller, 2020). For hyperprolactinaemia, meta-analytic evidence supports a contribution of DRD2 variants and CYP2D6 phenotypes to prolactin elevations during treatment with certain antipsychotics (Miura et al., 2016; Calafato et al., 2020). Similarly, systematic reviews have identified single-nucleotide polymorphisms (SNPs) associated with akathisia and other extrapyramidal symptoms, but replication is inconsistent, and effect sizes are modest (Nasyrova et al., 2023).

Overall, the genetic evidence suggests that adverse metabolic and endocrine effects of antipsychotics are influenced by both PK and PD genes, but that current markers lack sufficient predictive power to underpin routine prophylactic or stratified prescribing.

##### 3.1.3.4 Exploratory epigenetic and gene expression markers

Compared with antidepressants, the antipsychotic literature linking epigenetic or transcriptomic markers to *predictive*, clinically actionable outcomes is more limited and methodologically heterogeneous. A substantial proportion of the available work is mechanistic, cross-sectional, or not prospectively validated for decision-making, while clinically oriented syntheses continue to emphasise that actionable signals in antipsychotic precision prescribing cluster around PK/TDM-linked optimisation rather than receptor-level or other “omic” predictors of efficacy. For this reason, epigenetic and gene-expression findings are noted here as exploratory rather than developed as a parallel actionable evidence stream in the current hierarchy (Grant et al., 2025; Aldaz et al., 2021; de Leon, 2020; Teng et al., 2023).

##### 3.1.3.5 Special populations

In children and adolescents, the most consistent and reproducible antipsychotic PGx signal remains pharmacokinetic—particularly CYP2D6-linked exposure differences for risperidone—whereas evidence linking genotype to hard clinical outcomes (efficacy or tolerability endpoints) is less uniform. More broadly, where special-population data exist, they tend to reinforce a “PK-first” model of clinical usefulness rather than expanding the set of efficacy-predictive pharmacodynamic markers suitable for routine testing (Dodsworth et al., 2018; Maruf et al., 2021; Aldaz et al., 2021; de Leon, 2020).

#### 3.1.4 Mood stabilisers and lithium

A systematic review of 20 years of lithium pharmacogenetics synthesised data from numerous candidate-gene and genome-wide association studies (GWAS) and concluded that no consistent, replicable genetic predictor of lithium response has yet emerged (Pagani et al., 2019). Although several loci -particularly within genes involved in neuroplasticity, circadian regulation, and second messenger systems-have shown suggestive associations, these findings are heterogeneous and often cohort-specific. For other mood stabilisers, evidence is even sparser. Genetic predictors of lamotrigine or valproate response have been explored in small studies or as secondary analyses of trials, but no variant has reached the level of evidence seen for PK genes in antidepressant or antipsychotic therapy (Fabbri et al., 2017; Kato et al., 2015; Marshe et al., 2020).

#### 3.1.5 Other psychiatric and neuropsychiatric indications

Beyond MDD, schizophrenia and bipolar disorder, pharmacogenetic studies span a broad range of psychiatric and neuropsychiatric conditions, including autism spectrum disorder (ASD) and intellectual disability, obsessive–compulsive disorder (OCD), post-traumatic stress disorder (PTSD), substance use disorders (SUDs) and anxiety disorders. A systematic review of pharmacogenomic studies in intellectual disabilities and ASD reported numerous exploratory associations between PK and PD genes and psychotropic response or adverse events, but emphasised the paucity of high-quality trials and the absence of validated markers ready for clinical implementation (Yoshida et al., 2021). Pilot data suggest that pharmacogenetic profiling may help reduce psychotropic adverse events in ASD by avoiding extreme PK phenotypes or high-risk gene–drug combinations, but these findings require replication (de Miguel et al., 2023). Reviews in alcohol use disorders, OCD, and PTSD similarly describe a patchwork of the candidate-gene findings -often involving opioid receptor genes, serotonergic and dopaminergic variants- but no single marker with consistent predictive value across studies (Helton and Lohoff, 2015; Zai et al., 2021; Naß and Efferth, 2017; Baba et al., 2022).

### 3.1.5 Cross-cutting themes: polygenic risk, gene–environment and the limits of single-gene predictors

Several lines of evidence reinforce the conclusion that single variants are unlikely to capture the complexity of psychotropic treatment response. First, polygenic risk scores developed for psychiatric disorders explain only a modest proportion of liability and show limited correlation with treatment outcomes in current data sets (Grant et al., 2025; Müller et al., 2024). Second, the emerging “placebome” literature shows that genetic variation also influences placebo responsiveness, complicating the interpretation of drug-specific pharmacogenetic effects in clinical trials (Hall et al., 2015). Third, gene–environment interactions -such as smoking effects on CYP1A2, drug–drug–gene interactions involving P450 enzymes, and the impact of inflammatory or hormonal milieu-modulate genetic influences on pharmacokinetics and pharmacodynamics (Beunk et al., 2024; Stingl and Viviani, 2015; Na Takuathung et al., 2019; Thomas, , 2020).

Collectively, the genetic evidence base supports a hierarchy of markers:

1. *High-confidence, guideline-level PK markers* (CYP2D6, CYP2C19) for specific psychotropic drugs, especially certain antidepressants and antipsychotics.
2. *Moderate-confidence PD markers* (serotonin transporter/receptors, dopaminergic and neurotrophic genes) with small, inconsistent effects that are not currently recommended for routine testing.
3. *Exploratory epigenetic, transcriptomic, and polygenic markers* that enrich mechanistic understanding but remain far from clinical translation.

This hierarchy underpins the subsequent analysis of clinical integration, where the tension between solid but narrow PK evidence and broad but weak PD findings becomes central to evaluating when and how psychiatric pharmacogenomics should influence prescribing decisions.

### 3.2 Integration of psychiatric pharmacogenomics

#### 3.2.1 Landscape of PGx-guided vs. treatment-as-usual trials

Randomised trials and meta-analyses evaluating PGx-guided prescribing versus TAU form the core empirical basis for clinical integration. Five recent quantitative syntheses -including two broad meta-analyses of combinatorial pharmacogenomic testing (Brown et al., 2020; Skryabin et al., 2023), two focused on RCTs in MDD (Wang et al., 2023; Cheng et al., 2023), and one cumulative meta-analysis of guided versus unguided antidepressant therapy (Zhang et al., 2025)- consistently report small-to-moderate improvements in response and remission when prescribing is informed by multigene panels. However, these benefits are heterogeneous and context-dependent. Effect sizes are typically larger in trials with high prevalence of actionable gene–drug interactions at baseline, more stringent adherence to PGx recommendations, and more severe or treatment-resistant populations (Brown et al., 2020; Skryabin et al., 2023; Wang et al., 2023; Cheng et al., 2023; Zhang et al., 2025), while large pragmatic trials in unselected primary-care settings show more modest gains (Greden et al., 2019; Oslin et al., 2022).

#### 3.2.2 Meta-analytic evidence in depression

Meta-analyses pooling RCTs of PGx-guided antidepressant treatment in MDD converge on several points:

1. Response and remission: combinatorial PGx testing modestly increases the likelihood of response and remission compared with TAU, with the most consistent signal in treatment-resistant or highly pre-treated patients (Brown et al., 2020; Skryabin et al., 2023; Wang et al., 2023; Cheng et al., 2023; Zhang et al., 2025). In the largest RCT-focused meta-analysis to date, PGx-guided care was associated with higher response (week 8 OR 1.32, 95% CI 1.15–1.53; week 12 OR 1.36, 95% CI 1.15–1.62) and remission (week 8 OR 1.58, 95% CI 1.31–1.92; week 12 OR 2.23, 95% CI 1.23–4.04) compared with TAU in patients with MDD (Wang et al., 2023). The cumulative meta-analysis by Zhang et al. (2025) suggests that, as more trials accumulate, the overall estimate stabilises in the small-to-moderate range, with no indication that early positive studies were simply outliers (Zhang et al., 2025).
2. Symptom reduction and time to improvement: some syntheses report earlier or greater symptom reduction in guided arms (Brown et al., 2020; Skryabin et al., 2023; Wang et al., 2023), but these differences are less robust across sensitivity analyses and often attenuate when high-risk-of-bias studies are excluded (Wang et al., 2023; Cheng et al., 2023; Zhang et al., 2025).
3. Hospitalisation and healthcare utilisation: meta-analytic data on hospitalisation, emergency visits, or work functioning are sparse. Where reported, reductions favour guided treatment but are inconsistent and highly dependent on single large studies (Brown et al., 2020; Skryabin et al., 2023).
4. Heterogeneity and risk of bias: all meta-analyses underscore substantial between-study heterogeneity -driven by differences in panels, algorithms, blinding, sponsorship, and outcome definitions- and highlight a predominance of industry-funded trials (Brown et al., 2020; Skryabin et al., 2023; Wang et al., 2023; Cheng et al., 2023; Zhang et al., 2025). When analyses are restricted to more rigorous designs (adequate blinding, pre-specified primary outcomes, independent funding), effect sizes diminish, but generally do not disappear (Wang et al., 2023; Cheng et al., 2023; Zhang et al., 2025).

Overall, the meta-analytic literature supports the view that PGx-guided antidepressant prescribing improves outcomes on average, with typical pooled OR/RR estimates for response and remission in the ≈1.2–1.6 range, but with a modest absolute magnitude and considerable variability across settings (Brown et al., 2020; Wang et al., 2023; Cheng et al., 2023; Zhang et al., 2025).

#### 3.2.3 Key RCTs of PGx-guided antidepressant treatment

The GUIDED trial is the largest patient- and rater-blinded RCT of combinatorial pharmacogenomic testing in MDD (Greden et al., 2019). In this trial, patients were randomised to GeneSight-guided care or TAU; the primary outcome (symptom change at a pre-specified timepoint) did not differ significantly between arms, but several secondary outcomes (response, remission in the subset with gene–drug interactions) favoured guided treatment (Greden et al., 2019; Thase et al., 2019). Two independent meta-analyses that include GUIDED interpret these results as compatible with a real but modest benefit, particularly when testing reveals actionable gene–drug interactions and clinicians adhere to recommendations (Brown et al., 2020; Skryabin et al., 2023). However, the discordance between non-significant primary and positive secondary outcomes in GUIDED is one of the main reasons HTA bodies remain cautious (Health Quality Ontario, 2017). Other RCTs–such as the targeted PGx-guided trial by Bradley et al. (2018), the Canadian patient- and rater-blinded trial by Tiwari et al. (2022), and the PANDORA trial combining PGx with clinical algorithms–similarly report improvements in response or remission in guided arms, with effect sizes in broadly the same range as the meta-analytic estimates (Bradley et al., 2018; Tiwari et al., 2022; Minelli et al., 2021). A single-blind randomised study in depression also found better symptom trajectories when clinicians were provided with PGx results, although the design makes expectancy effects difficult to rule out (Shan et al., 2019).

Taken together, these trials illustrate a consistent pattern: PGx guidance rarely transforms outcomes, but it tends to shift probabilities modestly in favour of better response and tolerability, particularly in patients with a high burden of gene–drug conflicts.

The PRIME Care trial extended this work into a large U.S. Veterans Health Administration (VA) population with depression treated in routine practice (Oslin et al., 2022). In this pragmatic trial, clinicians in the PGx arm received reports on CYP2D6/CYP2C19 gene–drug interactions and recommendations, whereas the control arm followed usual prescribing practices. PRIME Care showed that PGx testing reduced prescribing of medications with predicted gene–drug interactions and that remission rates were modestly higher over follow-up in the guided arm (Oslin et al., 2022). However, absolute differences were minor, and not all timepoints reached statistical significance. These results support the real-world feasibility of integrating PGx into large healthcare systems, but also underline that structural and behavioural factors (clinician adherence, formulary constraints, patient preference) limit the translation of genetic information into large outcome gains.

#### 3.2.4 Special populations: older adults, adolescents, and long-term care

In older adults, a sub-analytical study of older MDD patients receiving combinatorial PGx-guided treatment, Forester et al. (2020) reported improved depressive outcomes compared with unguided care (Forester et al., 2020). These data, together with the broader late-life PGx literature (Marshe et al., 2020), suggest that older adults may particularly benefit from avoiding extreme metaboliser phenotypes and high-risk gene–drug combinations, given their higher vulnerability to adverse events.

A study that explored adolescent depression, a rater-blinded RCT, found that PGx-guided treatment was feasible and showed signals of improved outcomes relative to TAU, though sample size was modest and follow-up was short (Vande et al., 2022). A protocol for a double-blind RCT in paediatric anxiety disorders further illustrates growing interest in rigorous evaluation of PGx guidance in youth, but outcome data are not yet available (Strawn et al., 2021).

In long-term care facilities, an observational randomised implementation study integrating PGx into “individualised medication management” for depression, pain, and dementia showed improvements in clinical management and reductions in potentially inappropriate medications, although design limitations preclude firm causal inference (Dorfman et al., 2020).

These studies collectively indicate that PGx-guided prescribing is technically feasible and acceptable in vulnerable populations, but also that evidence remains thinner and more fragile than in working-age adults.

#### 3.2.5 Antipsychotic pharmacogenomics: early clinical trials

Clinical integration of PGx with antipsychotics is far less advanced than with antidepressants. The most informative trial is a randomised study in schizophrenia in which routine CYP2D6/CYP2C19 genotyping was compared with standard care, with antipsychotic drug persistence as the primary endpoint (Jürgens et al., 2020). Although genotyping led to some adjustments in dosing and drug choice, the trial did not demonstrate a large or unequivocal advantage of PGx-guided care on long-term persistence (Jürgens et al., 2020). Interpretation is complicated by heterogeneous medication regimens, limited power for individual gene–drug pairs, and the absence of structured algorithms comparable to those used in combinatorial antidepressant panels. Beyond this RCT, evidence for the clinical integration of antipsychotic PGx comes mainly from implementation programmes and observational studies that combine TDM with CYP2D6-guided dosing (Aldaz et al., 2021; de Leon, 2020). These suggest that PK-driven personalisation is plausible and may reduce adverse events, but robust RCT data on hard clinical outcomes (relapse, hospitalisation) are lacking.

#### 3.2.6 PGx as a complement to therapeutic drug monitoring

Pharmacogenomic testing and TDM address different clinical questions and are best viewed as complementary. PGx is most informative upstream, by indicating expected metabolic capacity (CYP2D6/CYP2C19 phenotype) and the risk of under- or overexposure at standard doses—particularly early in treatment and at metabolizer extremes. TDM, in contrast, measures actual exposure and integrates non-genetic determinants, including adherence, smoking-related CYP1A2 induction, inflammation, drug–drug interactions, and organ impairment. Consequently, while PGx can support initial drug/dose selection for high-confidence PK gene–drug pairs, it does not replace TDM when concentration-based individualization is required. This is particularly relevant for antipsychotics: de Leon emphasized that PGx alone is insufficient for dose individualization and that TDM remains essential, especially for clozapine, where CYP1A2 activity is strongly modified by smoking and inflammatory status (de Leon, 2020). A pragmatic approach is to use PGx to optimize initial selection/dosing and TDM to confirm exposure and troubleshoot non-response or adverse effects in higher-risk scenarios (Aldaz et al., 2021; de Leon, 2020).

### 3.3 From pharmacoeconomic analyses to real-life PGx utility in psychiatry

#### 3.3.1 Economic and utilisation outcomes in PGx-guided care

Several studies have examined whether PGx-guided prescribing translates into reduced costs or more efficient resource use. In PRIME Care, for example, the adjusted odds of remission at 24 weeks favoured PGx-guided care (OR 1.28, p = 0.02), but the absolute difference in remission was only 2.8 percentage points (approximate NNT ≈ 1/0.028 ≈ 36 over 24 weeks), illustrating how statistically significant relative gains can translate into modest absolute improvements (Oslin et al., 2022). Trial-based economic evaluations of genotype-specific dosing of tricyclic antidepressants and comparisons between PGx-based, phenotype-based, and standard dosing of nortriptyline indicate that PGx-guided strategies can be cost-effective when they prevent serious adverse events or reduce trial-and-error switching in high-risk patients (Ter et al., 2025; Vos et al., 2025). In psychiatric populations, *post hoc* economic analyses of combinatorial PGx trials in elderly patients and primary care report lower medication costs and fewer changes in treatment in guided arms (Jablonski et al., 2020; Brown et al., 2017), while a systematic review of cost-effectiveness across multiple CPIC-guided drugs (primarily non-psychiatric) finds that most studies favour PGx testing from a payer perspective (Morris et al., 2022). A recent Canadian microsimulation model for MDD further suggests that PGx-guided prescribing may be economically attractive at commonly accepted willingness-to-pay thresholds, particularly in recurrent or treatment-resistant depression (Ghanbarian et al., 2024). That said, economic benefits are highly sensitive to assumptions about test price, the prevalence of actionable genotypes, and the magnitude of clinical effect (Morris et al., 2022; Strawn et al., 2021; Dorfman et al., 2020; Jürgens et al., 2020; Ter et al., 2025; Vos et al., 2025; Jablonski et al., 2020; Brown et al., 2017; Ghanbarian et al., 2024). Health technology assessments explicitly caution against extrapolating favourable economic models from industry-sponsored trials and call for more independent, jurisdiction-specific evaluations (Health Quality Ontario, 2017).

#### 3.3.2 Implementation trials and health-system integration

Several implementation-focused projects shed light on how PGx testing performs when embedded in real clinical workflows, rather than in tightly controlled RCTs. Hospital and health-system programmes (e.g., UF Health’s personalised medicine programme) show that pre-emptive or reactive PGx testing can be integrated into electronic health records with decision support, and that psychiatric prescribing is among the areas where alerts for CYP2D6/CYP2C19 interactions are common (Cavallari et al., 2017). A recent scoping review of PGx implementation in hospital settings summarises a wide range of strategies–from pharmacist-driven consult services to automated alerts–and concludes that uptake is feasible but constrained by IT infrastructure, clinician education, and reimbursement (Wu et al., 2025). Within psychiatry-specific settings, analyses of CYP-GUIDES trial data demonstrate that providing PGx-based decision support in hospitalised depressed patients alters prescribing patterns and identifies a high prevalence of potential gene–drug interactions, particularly in ethnically diverse populations (Crutchley and Keuler, 2022; Ruaño et al., 2021). Parallel cohort studies in MDD document that the majority of patients harbour at least one predicted antidepressant gene–drug interaction, reinforcing the theoretical rationale for PGx-guided care even before clinical benefit is directly measured (Ramsey et al., 2021). Pragmatic projects in primary care and community pharmacies show that front-line clinicians and pharmacists can use PGx reports to adjust antidepressant or cannabis-related prescribing, but also that variability in panel content, report format and local expertise leads to inconsistent application of recommendations (Wu et al., 2025; Cavallari et al., 2017; Rollinson et al., 2020; Bradley et al., 2018).

#### 3.3.3 What the clinical integration data actually show

Based on the review of RCTs, meta-analyses, economic evaluations, and implementation studies, several robust conclusions emerged. First, antidepressant PGx panels add probabilistic value, not deterministic rules. Meta-analyses and large trials consistently suggest that PGx guidance yields incremental improvements in response/remission and modest reductions in gene–drug conflicts, particularly in patients with prior non-response or high burden of pharmacokinetic risk (Brown et al., 2020; Skryabin et al., 2023; Wang et al., 2023; Cheng et al., 2023; Zhang et al., 2025; Greden et al., 2019; Oslin et al., 2022; Thase et al., 2019; Bradley et al., 2018; Tiwari et al., 2022; Minelli et al., 2021; Shan et al., 2019). These gains are clinically relevant at the population level but fall short of a paradigm shift.

Second, context and implementation quality matter as much as biology. Trials with stronger effects typically combine: a high prevalence of actionable variants, structured algorithms, clinician adherence, and limited formulary constraints. Where any of these elements erode–e.g., in pragmatic settings with flexible care and incomplete uptake–effect sizes diminish, despite the same underlying genetics (Greden et al., 2019; Bradley et al., 2018; Tiwari et al., 2022; Dorfman et al., 2020; Crutchley and Keuler, 2022; Ruaño et al., 2021; Ramsey et al., 2021).

Third, evidence is strongest for antidepressants, weaker for antipsychotics, and sparse for other indications. For antipsychotics, single RCTs and multiple observational programmes suggest possible benefit of PK-guided dosing, but convincing RCT evidence for improved relapse or persistence is still lacking (Mao et al., 2023; Aldaz et al., 2021; Jürgens et al., 2020). For mood stabilisers and most other psychotropics, PGx integration remains largely inferential or exploratory (Fabbri et al., 2017; Stingl and Viviani, 2015; Marshe et al., 2020; Pagani et al., 2019).

Fourth, economic and structural arguments are promising but conditional. Economic analyses generally support the potential cost-effectiveness of PGx in selected psychiatric populations (Morris et al., 2022; Ter et al., 2025; Vos et al., 2025; Jablonski et al., 2020; Brown et al., 2017; Ghanbarian et al., 2024), yet these projections depend on assumptions about test pricing, effect size, and health-system efficiency that may not hold in all jurisdictions, particularly in low- and middle-income countries with different ancestry profiles and resource constraints (Morris et al., 2022; Koopmans et al., 2021; Ghanbarian et al., 2024).

In conclusion, the clinical integration data depict psychiatric pharmacogenomics as an incremental optimisation tool rather than a disruptive technology: useful when deployed thoughtfully in high-risk contexts, but insufficient on its own to guarantee robust, universal gains in psychiatric outcomes.

## 4 Discussion

### 4.1 Structural, economic and implementation barriers

#### 4.1.1 Fragmented and conservative guideline landscape

Current expert and guideline documents are cautiously positive but structurally conservative. The World Federation of Societies of Biological Psychiatry (WFSBP) consensus on pharmacogenomic testing in psychiatry explicitly recognises CYP2D6/CYP2C19 for several antidepressants and antipsychotics, yet stops short of recommending universal testing, emphasising limited effect sizes, heterogeneous evidence, and the need for context-specific implementation (Bousman et al., 2021). In parallel, the Dutch Pharmacogenetics Working Group (DPWG) issues gene–drug-specific dosing recommendations for psychotropics, often with higher granularity and stronger language for CYP2D6/CYP2C19 substrates, but it is a national rather than a global standard (Beunk et al., 2024). In addition, CPIC provides phenotype-based dosing recommendations for several SSRI/SNRI antidepressants (including CYP2C19-related guidance for citalopram/escitalopram), complementing consensus statements and jurisdiction-specific guideline bodies (Bousman et al., 2023). This patchwork of expert guidance–with some jurisdictions moving toward formalised dose–adjustment tables and others remaining non-committal–creates structural ambiguity for clinicians and healthcare systems. Where WFSBP-style documents emphasise the limits of evidence and DPWG-type guidelines provide concrete dosing actions, health systems can legitimately choose very different levels of investment in PGx infrastructure (Bousman et al., 2021; Beunk et al., 2024). HTA reports further reinforce this caution. The Ontario HTA on the GeneSight test concluded that, despite suggestive evidence of clinical benefit, inconsistent RCT outcomes, sponsorship bias, and unclear generalisability prevent firm recommendations for public reimbursement (Health Quality Ontario, 2017). This reinforces a structurally conservative stance: PGx is “interesting and potentially useful”, but rarely classified as essential.

#### 4.1.2 Economic uncertainty and payer hesitancy

Structural barriers are tightly linked to economic uncertainty. A systematic review of cost-effectiveness for CPIC-guided drugs across indications found that most economic models were favourable to PGx testing, but relied on assumed effect sizes and often industry-sponsored data, with limited psychiatric-specific analyses (Morris et al., 2022). Within psychiatry, trial-based evaluations of genotype-specific TCA dosing and nortriptyline optimisation suggest that PGx strategies can be cost-effective when they prevent serious toxicity or reduce protracted trial-and-error prescribing in difficult-to-treat depression (Ter et al., 2025; Vos et al., 2025). A Canadian simulation model for MDD illustrates this tension clearly: under plausible assumptions about test cost and clinical effectiveness, pre-emptive or early PGx testing can be economically attractive, but the model’s outputs are highly sensitive to the underlying effect size and prevalence of actionable genotypes (Ghanbarian et al., 2024). In other words, economic viability is conditional, not intrinsic, and depends heavily on local prices, formularies and population genetics. These uncertainties translate into payer hesitancy. In the absence of strong, universally positive RCT results and independent real-world cost data, many systems classify psychiatric PGx as an optional add-on rather than a reimbursed standard of care, thereby limiting scale and reinforcing structural inertia.

#### 4.1.3 Ancestry, allele frequency and equity of benefit

A deeper structural issue concerns global and ancestry-related variation in CYP2D6 and CYP2C19 allele frequencies. A large meta-analysis quantifying the worldwide distribution of CYP2D6/CYP2C19 phenotypes demonstrates striking differences between populations, with some ancestries showing much higher proportions of poor or ultrarapid metabolisers than the European reference groups upon which most trials and guidelines are based (Koopmans et al., 2021). For example, Koopmans et al. note that CYP2D6 ultrarapid metabolizers are relatively uncommon in Europeans (≈2–3%) but substantially more frequent in East-African populations (≈20–29%), while CYP2C19 poor metabolizers are more frequent in Asians (≈12%) than in Europeans (≈2%), implying that the yield and impact of PGx-guided dosing will differ across ancestries (Koopmans et al., 2021). A systematic review of pharmacogenomics in Sri Lanka underscores the consequences: allele frequencies and haplotypes in South Asian populations often diverge from those represented in commercial panels and CPIC/DPWG tables, raising doubts about the direct transferability of Western dosing recommendations (Ranasinghe et al., 2024). Similar concerns arise in many low- and middle-income countries where local PGx data are scarce, yet imported tests and algorithms are used without ancestry-specific validation (Koopmans et al., 2021; Ranasinghe et al., 2024). This mismatch is structurally important. If test panels are optimised for alleles common in European and North American populations, clinical yield will be lower, and misclassification risk higher in under-represented ancestries. For example, the estimated prevalence of CYP2D6 poor metabolisers is around 1%–2% in many East Asian populations but 5%–10% in Europeans, while CYP2C19 rapid and ultrarapid metabolisers can account for more than 25%–30% of individuals in some South and South-East Asian groups, implying that the same panel and dosing tables will have very different yields across ancestries (Koopmans et al., 2021). Health systems in LMICs may then rationally conclude that the cost per actionable result is unacceptable, reinforcing global inequities in access to precision psychiatry.

#### 4.1.4 Implementation infrastructure and workflow friction

Even when economic and genetic arguments are favourable, implementation infrastructure remains a major bottleneck. A recent scoping review of PGx implementation in hospital settings synthesised dozens of programmes and concluded that successful implementation depends on: laboratory capacity and validated genotyping methods; robust electronic health record (EHR) integration with clinical decision support (CDS); clear governance on result storage and re-use; and sustainable reimbursement and workflow alignment (Wu et al., 2025). Only a minority of hospitals meet all these conditions, and psychiatric prescribing competes for CDS bandwidth with oncology, cardiology, and anticoagulation, where effect sizes are often larger (Wu et al., 2025). The UF Health personalised medicine programme illustrates both the promise and the constraints: it has embedded PGx results and alerts into the EHR across multiple specialties, yet psychiatric modules are just one component of a broad genomic infrastructure requiring substantial upfront investment and ongoing informatics support (Cavallari et al., 2017). At the primary care level, a UK quality-improvement project deploying PGx in general practice showed that GPs can use PGx reports to adjust antidepressant therapy, but also highlighted variability in uptake, time pressure, and dependence on local champions (Bhimpuria, 2024). The IGNITE pragmatic trials network similarly demonstrates that real-world genomic implementation hinges on local leadership, IT integration, and tailored education rather than on evidence alone (Ginsburg et al., 2021).

#### 4.1.5 Commercial panels, health-system fit, and structural misalignment

There is a structural misalignment between commercial multigene panels and public health priorities. Panels are designed and marketed by companies with global ambitions, but reimbursement decisions are made by local payers whose priorities include transparency, reproducibility and independence from proprietary algorithms. HTA reports, including the GeneSight assessment, explicitly criticise opaque weighting schemes and limited disclosure of how genetic and clinical factors are combined into colour-coded recommendations (Health Quality Ontario, 2017). National and regional health systems are therefore faced with a choice: either accept commercial panels “as is”, with limited control over algorithm evolution, or invest in local panels and CDS pipelines that replicate or adapt the evidence base. Both options are expensive and organisationally demanding, which explains why many systems default to minimal, gene-by-gene testing for a small set of CPIC/DPWG Level A interactions, rather than full psychiatric PGx panels.

In sum, structural and implementation barriers are not primarily scientific; they arise from misalignment between modest, probabilistic clinical gains and the heavy infrastructural, economic and organisational demands of genomic integration.

### 4.2 Educational and ethical challenges

#### 4.2.1 Knowledge gaps, over-trust, and the “black box” problem

Even when infrastructure exists, psychiatric pharmacogenomics runs into pervasive educational challenges. Early commentaries on commercial decision-support tools emphasised that many psychiatrists overestimate the determinism of PGx reports and underestimate their limitations, particularly around PD markers with weak evidence (Bousman and Hopwood, 2016). A systematic evaluation of commercial psychiatric PGx tests showed substantial variation in allele coverage, phenotype calling and result reporting, especially for CYP2D6/CYP2C19, and concluded that discordant outputs across tests risk confusing clinicians who lack deep pharmacogenetic training (Bousman et al., 2017). This creates a paradoxical combination of under- and over-trust: some clinicians ignore PGx entirely, while others treat panel outputs as quasi-authoritative, despite the modest and context-dependent effect sizes demonstrated in RCTs and meta-analyses. Health technology assessments, such as the GeneSight HTA, explicitly warn against using PGx results to override clinical judgement or to justify abrupt medication changes without considering past response, comorbidities and patient preference (Health Quality Ontario, 2017). Educational initiatives lag behind commercial penetration. Implementation reviews and hospital experience reports repeatedly identify clinician education and genomic literacy as central barriers: many prescribers are unclear about how to interpret metaboliser status, how to reconcile discordant gene–drug recommendations, and when not to order tests at all (Wu et al., 2025; Ginsburg et al., 2021; Cavallari et al., 2017).

#### 4.2.2 Ancestry, panel design and distributive justice

Ethical concerns about equity and justice run parallel to the structural issues described above. Commercial and academic tests are typically optimised for alleles prevalent in European-ancestry populations; systematic work quantifying global CYP2D6/CYP2C19 variation and national reviews from South Asia and other regions consistently show that many common non-European alleles are either underrepresented or absent from standard panels (Koopmans et al., 2021; Ranasinghe et al., 2024). This raises several ethical questions: Are we systematically delivering lower-quality PGx information to patients from underrepresented ancestries? Does the use of partially mis-specified panels increase the risk of erroneous reassurance (e.g., “no interaction” reports that simply reflect missing alleles)? Should payers in LMICs invest in PGx when the underlying panels have not been validated in their populations?

National reviews of PGx landscapes in settings such as Sri Lanka highlight the risk that PGx could widen rather than narrow global health inequities, if wealthy systems benefit from ancestry-matched panels and robust implementation, while resource-constrained settings either lack access or use suboptimal tests (Ranasinghe et al., 2024).

#### 4.2.3 Regulation, consent and the scope of data reuse

Implementation reviews in hospitals underscore that PGx results, once generated, are often stored and reused across specialties and over a patient’s lifetime. This creates a pragmatic “incidental findings” problem in a PGx sense: results generated for one prescribing decision may later become clinically relevant for other drugs, other specialties, or after guideline/allele reclassification. Accordingly, governance should specify secondary-use boundaries, re-interpretation/update procedures, and (where feasible) recontact expectations. (Wu et al., 2025; Cavallari et al., 2017). This raises complex consent and governance questions: Do patients understand that a test ordered in psychiatry may later influence prescribing in oncology or cardiology? How should health systems handle requests to delete or restrict genomic data? What level of recontact obligation exists when phenotype interpretation is revised (e.g., when new alleles are reclassified)? In pediatric psychiatry, these consent and data-lifespan issues are amplified because results may be reused for decades; therefore, testing is most defensible when anchored in high-evidence PK gene–drug pairs and clear clinical questions (e.g., tolerability risk or dosing) rather than broad “best-drug” claims (Aldrich et al., 2019; Poweleit et al., 2019; Amitai et al., 2016; Honeycutt et al., 2024; Strawn et al., 2021).

A parallel literature in forensic and legal medicine points to additional ethical frictions. Pharmacogenetic information can, in principle, be used to reinterpret adverse drug reactions, intoxications, or deaths in medico-legal investigations, and some have proposed PGx as a tool to refine responsibility assessments in cases involving psychotropic drugs (Di Nunno et al., 2021). While this is still a niche application, it illustrates how PGx data may be drawn into legal processes far beyond the original psychiatric indication. These issues argue for explicit, layered consent models, clearer boundaries on secondary use, and robust governance structures–none of which are yet standard in routine psychiatric practice.

#### 4.2.4 Commercial interests, transparency and conflict of interest

Commercialisation introduces further ethical complexity. Commentaries and empirical analyses of psychiatric PGx tests emphasise the intertwining of evidence generation and marketing, with many RCTs and economic evaluations sponsored by test manufacturers (Bousman and Hopwood, 2016; Bousman et al., 2017; Health Quality Ontario, 2017; Jablonski et al., 2020; Brown et al., 2017). Although sponsorship does not invalidate results, it heightens the need for independent replication and transparent reporting of negative or equivocal trials. The opacity of proprietary algorithms compounds this concern. If clinicians cannot inspect how gene–drug pairs are weighted, or how PD markers with weak evidence are incorporated alongside robust PK markers, it becomes difficult to judge whether a panel’s recommendations are evidence-proportionate or skewed by commercial priorities. Health technology assessments and national reviews repeatedly call for greater algorithmic transparency as a precondition for public reimbursement (Health Quality Ontario, 2017; Ranasinghe et al., 2024).

#### 4.2.5 System-level ethics: opportunity costs and implementation priorities

At the health-system level, the key ethical question is not whether PGx “works” in a narrow sense–meta-analyses suggest it does, modestly–but whether investing in psychiatric PGx yields a greater marginal benefit than competing interventions (psychotherapy access, collaborative care, social interventions). Economic modelling and implementation networks, such as IGNITE, show that genomic programmes demand substantial infrastructural investment, and the opportunity cost of those resources is non-trivial (Wu et al., 2025; Ginsburg et al., 2021; Cavallari et al., 2017; Ghanbarian et al., 2024). From an ethical standpoint, prioritising PGx ahead of basic access to effective psychotherapies or medication adherence support may be difficult to justify in under-resourced settings. National primary-care QI work demonstrates that even in high-income countries, implementing PGx competes with more mundane but impactful quality-improvement targets (blood pressure control, diabetes care, vaccination) (Bhimpuria, 2024).

### 4.3 Toward a more ethically coherent integration

Taken together, the educational and ethical literature suggests several guardrails for responsible integration of psychiatric PGx. For example, the principle of *epistemic humility and proportionality*. Training should explicitly convey the modest, probabilistic nature of PGx benefits, preventing both nihilism (“it changes nothing”) and genetic determinism (“the test decides”) (Bousman et al., 2021; Bousman and Hopwood, 2016; Bousman et al., 2017; Health Quality Ontario, 2017).

Also, *the ancestry-aware panel design and validation.* Population-genetic data and national reviews should inform which tests are deployed in which settings, with a bias toward panels that include alleles common in the local population and have been locally validated (Koopmans et al., 2021; Ranasinghe et al., 2024).

*Transparent, auditable decision support* needs to be highlighted in psychotropics PGx. Public or semi-public documentation of algorithms, allele coverage and evidence grading is ethically preferable to opaque commercial black boxes, particularly when public funds are involved (Bousman et al., 2017; Health Quality Ontario, 2017; Wu et al., 2025; Cavallari et al., 2017).

*Robust consent and governance frameworks* are required for this field. Layered consent, clear rules on data reuse, and explicit policies for medico-legal access to PGx data are needed to prevent gradual function creep into forensic or discriminatory uses (Wu et al., 2025; Cavallari et al., 2017; Di Nunno et al., 2021; Smith et al., 2019).

Without these measures, psychiatric pharmacogenomics risks becoming a commercially driven, inequitable and partially misunderstood technology: scientifically sound in its core PK insights, but ethically and educationally under-anchored.

## 5 Limitations of this review and future directions

Several limitations of this narrative review must be acknowledged. First, the design is intentionally non-systematic. Although we built on multiple systematic reviews, meta-analyses, consensus statements, and HTAs (Fornaguera and Miarons, 2025; Grant et al., 2025; Fabbri and Serretti, 2020; Bousman et al., 2021; Beunk et al., 2024; Brown et al., 2020; Zhang et al., 2025; Health Quality Ontario, 2017; Morris et al., 2022; Kato et al., 2015), we did not conduct a *de novo* systematic search with predefined inclusion/exclusion criteria, dual screening, or formal risk-of-bias assessment. Because several of the included meta-analyses and umbrella syntheses likely draw on partially overlapping primary trials, we did not formally quantify study overlap, and some duplication of evidence across secondary sources is therefore possible. Selection and interpretation are therefore susceptible to author-level bias, including over-representation of studies that have become influential in the field (for example, GUIDED, PRIME Care, and specific combinatorial panels) and under-representation of negative or unpublished trials.

Second, the evidence base itself is uneven. Antidepressant pharmacogenomics in MDD -especially CYP2D6/CYP2C19-guided prescribing and multigene panels-dominates both genetic and clinical integration data (Fornaguera and Miarons, 2025; Grant et al., 2025; Brown et al., 2020; Skryabin et al., 2023; Wang et al., 2023; Cheng et al., 2023; Zhang et al., 2025). In contrast, antipsychotic PGx, mood stabilisers, and most other psychiatric indications are informed largely by smaller meta-analyses, observational cohorts, and mechanistic studies, with few RCTs and almost no large, independent implementation trials (Santoro et al., 2016; Fabbri et al., 2017; Kato et al., 2015). As a result, the strength of inference varies considerably across drug classes, even within this single review.

Third, our synthesis is anchored primarily in English-language publications from high-income countries. Although we explicitly included work on ancestry variation and national PGx landscapes in LMICs (Koopmans et al., 2021; Rollinson et al., 2020; Ranasinghe et al., 2024), the underlying literature is heavily skewed toward European-ancestry populations and high-resource health systems. This limits the external validity of many conclusions for settings with different genetic backgrounds, prescribing patterns and infrastructural constraints.

Fourth, we did not attempt a formal quantitative integration of economic data. Cost-effectiveness models differ in perspective (payer versus societal), time horizon, costing assumptions and outcome measures, and often rely on effect sizes from industry-sponsored trials (Health Quality Ontario, 2017; Morris et al., 2022; Ter et al., 2025; Vos et al., 2025; Jablonski et al., 2020; Brown et al., 2017; Ghanbarian et al., 2024). Any narrative summary of such heterogeneous models risks oversimplifying the conditional nature of economic attractiveness.

Fifth, the ethical and educational analysis draws heavily on implementation studies, HTAs and expert commentaries (Bousman et al., 2021; Beunk et al., 2024; Bousman and Hopwood, 2016; Bousman et al., 2017; Health Quality Ontario, 2017; Morris et al., 2022; Koopmans et al., 2021; Cavallari et al., 2017; Kato et al., 2015; Kim et al., 2021). These sources are invaluable for highlighting real-world frictions but are not immune to their own biases—whether towards enthusiasm (in implementation networks) or caution (in HTAs). There is a relative paucity of empirical work on patient perspectives, consent comprehension, perceptions of equity and the long-term psychosocial impact of PGx testing in psychiatry.

Finally, the field is moving rapidly. New RCTs, guidelines and implementation programmes are emerging, and allele interpretation frameworks continue to evolve. Any static synthesis risks being quickly outpaced by ongoing developments in panel design, regulatory oversight and reimbursement policies. Consequently, the conclusions offered here should be read as a snapshot of the evidence up to mid-2025, rather than as a definitive or exhaustive account.

Future research should move beyond single-marker candidate studies and small, industry-sponsored trials. Large, independently funded RCTs and pragmatic implementation studies are needed in antipsychotic and mood stabiliser pharmacogenomics, in diverse ancestry groups and in LMIC health systems. Economic evaluations should incorporate real-world effect sizes, local cost structures and opportunity costs relative to other mental-health investments (Health Quality Ontario, 2017; Morris et al., 2022; Colle et al., 2015; Kim et al., 2021). The integration of epigenetics, exosomes, protein interactions, microbiome, and nutrigenomics data in future studies on PGx would increase the chances of achieving actual individualised treatment in psychiatry, targeting not only frequent psychopathological entities, but also less frequent and still functionally severely impairing disorders (Manea et al., 2024; Truong et al., 2025; Shaman, 2024; Vasiliu, 2024; Smith and Woodside, 2016; Kulisevsky et al., 2025). Ethical scholarship must be grounded in empirical data on patient preferences, consent comprehension, and perceived fairness, not solely in expert opinion.

If these directions are pursued, psychiatric pharmacogenomics is likely to consolidate its role as a targeted optimisation tool—particularly for high-risk, treatment-resistant patients—embedded within broader, biopsychosocial models of care. If they are neglected, the field risks remaining a commercially driven niche: scientifically credible in its PK core, but structurally fragmented, educationally fragile and ethically contested.

## 6 Conclusion

Psychiatric pharmacogenomics occupies a nuanced intersection between relatively strong but narrow pharmacokinetic evidence, weaker and heterogeneous pharmacodynamic findings, and substantial implementation and ethical constraints. The most clinically actionable data concern CYP2D6 and CYP2C19 variants for certain antidepressants and, to a lesser extent, antipsychotics, which reliably predict serum levels and adverse effects and show modest associations with response and remission. Multigene panels extend this evidence base and, in RCTs and meta-analyses, yield small-to-moderate improvements in outcomes in major depressive disorder, particularly in treatment-resistant patients. In contrast, most pharmacodynamic, epigenetic, and transcriptomic markers remain exploratory and insufficient for guideline-level use, and evidence outside depression is limited. Implementation studies indicate that clinical benefit depends heavily on contextual factors such as decision-support infrastructure, clinician uptake and reimbursement, without which biological advantages are substantially attenuated.

Ethically, psychiatric pharmacogenomics raises concerns that extend beyond clinical efficacy, particularly regarding equity, transparency, and governance. Ancestry-related bias in current panels, proprietary algorithms, industry influence, and the lifelong reuse of genetic data collectively complicate informed consent, trust, reimbursement, and medico-legal accountability.

Taken together, these strands point toward a pragmatic, proportionate model of integration rather than a binary embrace or rejection of psychiatric pharmacogenomics. In the near term, the most defensible strategy is:

*To prioritise guideline-level PK markers (particularly CYP2D6/CYP2C19) for clearly defined psychotropic gene–drug pairs* where evidence is strong and dosing recommendations are explicit.

*To deploy multigene panels selectively*, focusing on patients with treatment-resistant depression, complex polypharmacy or high risk of adverse events, and embedding panels within transparent, auditable CDS frameworks.

*To design ancestry-aware implementation pathways*, ensuring that allele coverage and phenotype calling reflect local population genetics, and that underrepresented groups are not systematically disadvantaged.

*To invest in education and governance at least as much as in testing itself*, emphasising probabilistic interpretation, data stewardship and realistic expectations among clinicians, patients and payers.

## Supplementary material

SUPPLEMENTARY FIGURE 1 PRISMA-style flow schematic of evidence identification and theme-driven mapping. Records were identified through database searches in PubMed/MEDLINE (n = 58), Scopus (n = 105), and Web of Science/Clarivate (n=99) (total n = 262). After de-duplication, 129 unique records constituted the initial seed set for the narrative synthesis. Additional records were incorporated iteratively through backward/forward citation tracking and targeted theme-driven searches to support the three focal domains of the review.
