# Supplementary figures and images for: Psychiatric pharmacogenomics: from genetic evidence to clinical integration – structural, educational, and ethical challenges
Source: Front Pharmacol. 2026 Apr 7;17:1775522. doi: 10.3389/fphar.2026.1775522 (PMC13096094; doi:10.3389/fphar.2026.1775522)

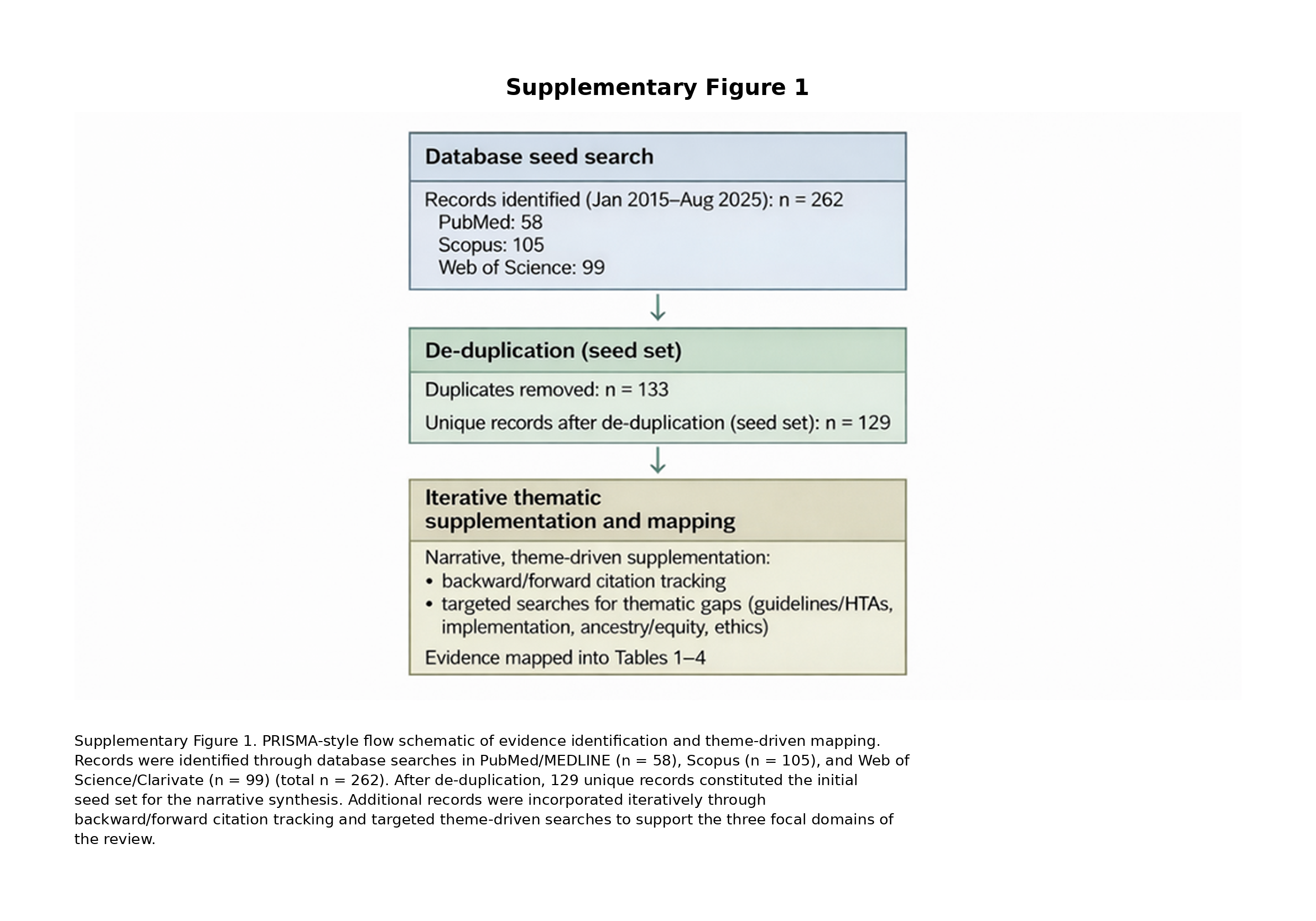

Supplement: Supplementary file 1 [file Image1.tiff]
